# Dually Cross-Linked Core-Shell Structure Nanohydrogel with Redox–Responsive Degradability for Intracellular Delivery

**DOI:** 10.3390/pharmaceutics13122048

**Published:** 2021-11-30

**Authors:** Siyuan Deng, Maria Rosa Gigliobianco, Emin Mijit, Marco Minicucci, Manuela Cortese, Barbara Campisi, Dario Voinovich, Michela Battistelli, Sara Salucci, Pietro Gobbi, Giulio Lupidi, Giorgia Zambito, Laura Mezzanotte, Roberta Censi, Piera Di Martino

**Affiliations:** 1 School of Pharmacy, University of Camerino, Via S. Agostino 1, 62032 Camerino, Italy; siyuan.deng@unicam.it (S.D.); manuela.cortese@unicam.it (M.C.); giulio.lupidi@unicam.it (G.L.); 2 Percuros B.V., Zernikedreef 8, 2333 CL Leiden, The Netherlands; m.gigliobianco@percuros.nl; 3 Physics Division, School of Science and Technology, University of Camerino, Via Madonna delle Carceri 9, 62032 Camerino, Italy; emin.mijiti@unicam.it (E.M.); marco.minicucci@unicam.it (M.M.); 4 Department of Economic, Business, Mathematic and Statistical Sciences, University of Trieste, 34127 Trieste, Italy; campisi@units.it; 5 Department of Chemical and Pharmaceutical Science, University of Trieste, P. le Europa 1, 34127 Trieste, Italy; vojnovic@units.it; 6 Institute of Morphological Sciences, University of Urbino, Via Ca’ le Suore 2, 61029 Urbino, Italy; michela.battistelli@uniurb.it (M.B.); pietro.gobbi@uniurb.it (P.G.); 7 Cellular Signalling Laboratory, Department of Biomedical and Neuromotor Sciences (DIBINEM), University of Bologna, 40126 Bologna, Italy; sara.salucci@unibo.it; 8 Department of Radiology and Nuclear Medicine, Erasmus Medical Center, 3015 GD Rotterdam, The Netherlands; g.zambito@erasmusmc.nl (G.Z.); l.mezzanotte@erasmusmc.nl (L.M.); 9 Dipartimento di Farmacia, Università “G. D’Annunzio” Chieti e Pescara, Via dei Vestini, 1, 66100 Chieti, Italy; piera.dimartino@unicam.it

**Keywords:** nanocapsule, macromolecule, peroxidase-like enzymatic activity, drug delivery system, cancer immunotherapy, factor influence study

## Abstract

A redox-responsive nanocarrier is a promising strategy for the intracellular drug release because it protects the payload, prevents its undesirable leakage during extracellular transport, and favors site-specific drug delivery. In this study, we developed a novel redox responsive core-shell structure nanohydrogel prepared by a water in oil nanoemulsion method using two biocompatible synthetic polymers: vinyl sulfonated poly(*N*-(2-hydroxypropyl) methacrylamide mono/dilactate)-polyethylene glycol-poly(*N*-(2-hydroxypropyl) methacrylamide mono/dilactate) triblock copolymer, and thiolated hyaluronic acid. The influence on the nanohydrogel particle size and distribution of formulation parameters was investigated by a three-level full factorial design to optimize the preparation conditions. The surface and core-shell morphology of the nanohydrogel were observed by scanning electron microscope, transmission electron microscopy, and further confirmed by Fourier transform infrared spectroscopy and Raman spectroscopy from the standpoint of chemical composition. The redox-responsive biodegradability of the nanohydrogel in reducing environments was determined using glutathione as reducing agent. A nanohydrogel with particle size around 250 nm and polydispersity index around 0.1 is characterized by a thermosensitive shell which jellifies at body temperature and crosslinks at the interface of a redox-responsive hyaluronic acid core via the Michael addition reaction. The nanohydrogel showed good encapsulation efficiency for model macromolecules of different molecular weight (93% for cytochrome C, 47% for horseradish peroxidase, and 90% for bovine serum albumin), capacity to retain the peroxidase-like enzymatic activity (around 90%) of cytochrome C and horseradish peroxidase, and specific redox-responsive release behavior. Additionally, the nanohydrogel exhibited excellent cytocompatibility and internalization efficiency into macrophages. Therefore, the developed core-shell structure nanohydrogel can be considered a promising tool for the potential intracellular delivery of different pharmaceutical applications, including for cancer therapy.

## 1. Introduction

In the past decades, the development of novel drug delivery systems has been attracting increasing interest to promote the progress of medical diagnosis and treatment. Enormous polymeric nanoscale particles have been studied as drug carriers, including manganic inorganic nanoparticles [1], noisome [2,3], nanomotor [4], micelle [5], solid nanoparticles [6,7], and nanohydrogels [8], among others. Nanohydrogels, as hydrophilic nanoparticles, have been widely studied to meet particular and advanced applications, such as easy parenteral administration and targeted, intracellular, or systemic drug delivery [9]. Nanohydrogels integrate advantages from both hydrogel systems and nanoparticle systems. Different from the most commonly studied solid polymeric nanoparticles, nanohydrogels composed of water-swollen and cross-linked biopolymers, exhibit inherent biocompatibility, hydrophilicity, tissue-like mechanical properties, and high porosity. These characteristics make them particularly suitable for the encapsulation of macromolecular biotherapeutics, for example cytokine IL-2 [10] and peptide epitopes [11], to contribute to cancer immunotherapy. Furthermore, their nanoscale dimensions coupled with their hydrophilicity make them suitable for systemic administration and prolonged circulation time in the blood stream [12].

Several approaches have been used for the preparation of nanohydrogels as a drug delivery system. The formulation procedure, typically, consists of two decisive steps: monomer polymerization and nanohydrogel formulation by cross-linking. The polymer chains can be synthesized simultaneously with the formulation of nanohydrogel [13], or polymer precursors are formed beforehand by polymerization of monomers, and nanohydrogels are obtained from cross-linking reactions among the chains of polymer precursors subsequently [14]. According to the cross-linking methods, nanohydrogels can be divided into physically and chemically cross-linked nanohydrogels. Physically cross-linking methods refer to self-assembled structures of polymer precursors via physical interactions, generally including hydrophobic bonding, hydrogen and ionic interactions [15,16]. However, physically cross-linked nanohydrogels present low mechanical properties and stability, especially under in vivo conditions, due to the weak physical forces holding together the polymeric chains [17]. In contrast, chemically cross-linked nanohydrogel delivery systems usually provide superior mechanical properties because of relatively strong covalent bonding. The most widely used method for chemical cross-linked nanohydrogel preparation is heterogeneous polymerization, including precipitation polymerization and nanoemulsion polymerization [18,19,20], which allow the preparation of nanohydrogels with different structures, such as a core-shell nanohydrogel [21,22], nanosphere [23,24,25] or nanohydrogel with a hollow cavity [26,27,28]. A number of cross-linking approaches, including click chemistry [29,30], Schiff-base reaction [31,32], photo-induced cross-linking [33], and enzymatic cross-linking [34] have been studied to formulate a nanohydrogel from polymer precursors.

To act as an ideal drug carrier, the nanohydrogel must guarantee drug stability during the delivery period, but also allow for drug release at the target site. Therefore, many stimuli-responsive nanohydrogels have been designed and developed as controlled drug delivery systems. The physical or chemical structure of these intelligent nanohydrogels can effectively respond to environmental stimuli such as temperature [28,35,36], pH [37,38,39], redox potential [40,41,42], ionic strength [43,44] or the combination of these factors [45,46,47,48], to achieve the purpose of targeted and controlled drug release. In particular, redox responsive nanohydrogels have been intensively investigated as potential intracellular delivery systems [49]. Disulfide crosslinking has been introduced into nanohydrogels for the “on-demand” release of drugs into target sites characterized by high concentrations of reducing agents such as glutathione (GSH) [50]. It is well known that the concentration of GSH in the intracellular or tumor environment (2–10 mM) is higher than the concentration in the extracellular environment (2–10 μM) [51,52]. It has been demonstrated by different studies that redox responsive nanohydrogels cross-linked by disulfide bonds can effectively protect the payload, including protein drugs [53], gene therapeutic agents [54] or small molecular anticancer drugs [55] during circulation in the bloodstream, and specifically release the payload at the target site. Therefore, redox responsive nanohydrogels have recently attracted increasing attention as intracellular delivery systems for improving the therapeutic effect for different diseases, in particular for cancer treatment. It has been shown that intracellular delivery of antigenic peptide into the antigen-presenting cell, i.e., dendritic cells or macrophages, can effectively improve the therapeutic efficacy of a peptide/protein based vaccine for cancer immunotherapy [56,57].

In our previous studies, a novel injectable thermosensitive hydrogel was developed as a promising candidate for tissue engineering and controlled drug delivery applications. This bicomponent hydrogel comprised thiolated hyaluronic acid (HA-SH) and a triblock copolymer of poly(*N*-(2-hydroxypropyl) methacrylamide mono/di lactate)-polyethylene glycol partly modified with vinyl sulfone moieties (Trib-sulf) [58]. This hydrogel system was designed to cross-link via a dual mechanism, i.e., thermal gelation at 37 °C of the thermosensitive poly(*N*-(2-hydroxypropyl) methacrylamide mono/di lactate segments of Trib-sulf, and a simultaneous Michael addition reaction between thiol groups of HA-SH and vinyl sulfone groups of Trib-sulf. In addition to favorable in vitro and in vivo biocompatibility and biodegradability, this hydrogel system also presented a significant anti-inflammatory effect in an osteoarthritis mouse model that was attributed to the controlled and sustained release of hyaluronic acid (HA) during hydrogel degradation [58,59,60].

More recently, a new redox sensitive nanohydrogel based on HA-SH was developed in our group as a systemic delivery system for oncolytic viruses (OVs) in cancer immunotherapy [61]. The nanohydrogel successfully encapsulated the OVs, maintained their biological activity, and released them in a tumor-mimicking environment due to its cytocompatibility, hydrophilicity and redox responsiveness.

The aim of the present study is to further develop the previously designed redox-sensitive nanohydrogel technology into a novel core-shell structure nanohydrogel for extended pharmaceutical and biomedical applications. This core-shell structure nanohydrogel, also called nanocapsule (NanoC), was designed and developed using HA-SH as a hydrophilic core and a Trib-sulf copolymer as a thermosensitive shell. This NanoC displayed a redox sensitive disulfide cross-linked core interfacially polymerized via Michael addition to the thermosensitive shell. The thermosensitive shell presented hydrophobicity at a temperature higher than the lower critical solution temperature (LCST) of Trib-sulf, which is 31 °C. Therefore, it can reduce the undesirable and premature diffusion of a water-soluble payload from the hydrophilic core of the NanoC immediately upon administration. Additionally, the thermosensitive shell can be considered as a potential loading location for a hydrophobic payload and/or be modified by specific targeting ligands for targeted drug delivery. The stability of NanoC in nonreducing and reducing environments was determined by particle size comparison between phosphate buffered saline (PBS) supplemented with 10 mM glutathione (GSH) as a reducing agent, to mimic the intracellular or tumor environment, and PBS without GSH to mimic the extracellular environment. In the reducing environment, NanoC exhibited a swelling behavior during the first 2 to15 h due to the cleavage of the disulfide bonds of the core and the stable Michael addition cross-linked shell, and a subsequent rapid degradation due to excessive hydration that caused nanohydrogel collapse. Meanwhile, the particle size of the NanoC kept constant in the physiological condition. Three proteins, cytochrome C (CC), horseradish peroxidase (HRP) and bovine serum albumin (BSA) differing in molecule weight (Mw, Mw_CC_ = 12.5 kDa, Mw_HRP_ = 44 kDa and Mw_BSA_ = 64 kDa) were loaded into the NanoC as model drugs to study the loading capacity and redox-responsive release behavior of the developed NanoC. A nanohydrogel with a nanosphere (NanoS) structure composed of a disulfide cross-linked core without the thermosensitive shell was prepared as a comparison. Additionally, the cytocompatibility and internalization of the NanoC was assessed on the RAW 264.7 macrophages cell line.

## 2. Materials and Methods

### 2.1. Materials

Unless stated otherwise, chemicals were purchased from Sigma Aldrich (Schnelldorf, Germany) and used as received. Sodium HA was purchased from Lifecore, Biomedical (Chaska, MN, USA). A polyethylene oxide/polyethylene glycol calibration kit for gel permeation chromatography (GPC) was purchased from Agilent Technologies (Santa Clara, CA, USA). A micro bicinchoninic acid (BCA) protein assay kit and lactate dehydrogenase (LDH) cytotoxicity assay kit were purchased from Thermo Fisher Scientific (Waltham, MA, USA). Fluorescent stains 4′,6-diamidino-2-phenylindole (DAPI) and LysoTracker^TM^ Deep Red were purchased from Vector Laboratories (Burlingame, CA, USA) and Thermo Fisher Scientific (Waltham, MA, USA), respectively. Additionally, 4-(dimethylamino) pyridinium-4-toluenesulfonate (DPTS) was previously synthesized in laboratory according to the procedure described by Moore et al. [62]. Ultrapure water was produced in the laboratory using a Milli-Q^®^ system (Merck Millipore, Darmstadt, Germany) with a resistivity at 18.2 MΩ-cm.

### 2.2. Synthesis and Characterization of Vinyl Sulfonated Triblock Copolymer (Trib-Sulf) and Thiolated Hyaluronic Acid (HA-SH)

See Supplementary Data. The synthesis route of Trib-sulf and HA-SH are described in Figures S1 and S2, respectively.

### 2.3. Preparation of the Nanocapsule (NanoC) and Nanosphere (NanoS)

**Figure 1:**
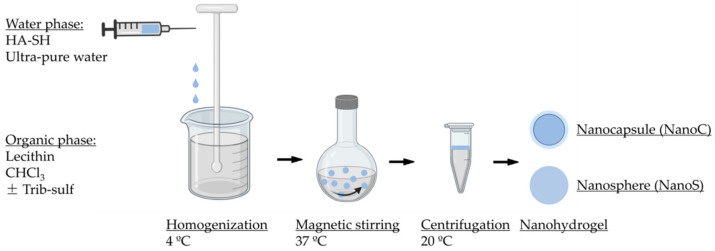
Schematic of nanocapsule (NanoC) and nanosphere (NanoS) formulation procedure.

The NanoC was prepared by the water in oil (W/O) nanoemulsion method using high speed homogenization (Ultra-Turrax^®^ T25 digital, IKA, Staufen, Germany). The general formulation procedure is described as follow (Figure 1). The water phase was prepared by dissolving HA-SH in ultrapure water at a concentration of 5% *w/v* and added dropwise into an organic phase prepared by dissolving Trib-sulf in chloroform (CHCl_3_) supplemented with lecithin (2.5% *w*/*v*) as a surfactant. During the addition of the water phase into the organic phase, high speed homogenization was maintained while keeping the sample at 4 ± 1 °C using an ice bath to avoid rapid evaporation of the organic phase and allowing the permeation of the Trib-sulf copolymer into the water phase. Subsequently, the nanoemulsion was incubated at a temperature of 37 °C and kept under magnetic stirring at 380 rpm to provide sufficient time for chemical crosslinking. Eventually, the NanoC was collected under centrifugation (high-speed microcentrifuge, D3024R, Scilogex, Rocky Hill, CT, USA) at 8000 rpm for 20 min. The upper water phase containing NanoC was collected and washed with CHCl_3_ three times to remove surfactant and excess unreacted Trib-sulf copolymer. The residual CHCl_3_ was removed under vacuum at room temperature and the nanohydrogels were isolated by lyophilization (Freezone, Labconco, Kansas, MO, USA) in the presence of 7% *w*/*v* sucrose as a lyoprotectant.

An additional series of NanoS units formulated in the absence of the Trib-sulf copolymer were prepared as a control formulation to verify the influence of the Trib-sulf shell on the stability and drug release behavior of the core-shell structure nanohydrogel.

### 2.4. Factor Influence Study for NanoC Preparation

To achieve particle size within the range of 200–300 nm and a good size distribution suitable for further application as a delivery system for systemic administration, an experimental design was applied for controlling the particle size and the polydispersity index (PDI) of the NanoC. According to preliminary studies, three independent variables were selected: the homogenization speed, the homogenization time, and the cross-linking time (Table 1). A three-level full factorial design (3^3^) was considered for evaluating the influence of such experimental factors (the independent variables) on the nanoparticle size and PDI of prepared NanoC (the dependent variable or experimental response). The factorial design and the experimental plan (Table 2) were obtained using NemrodW software (NemrodW SAS, Marseille, France).

### 2.5. Dynamic Light Scattering (DLS)

The particle size, PDI and zeta potential of NanoC and NanoS were characterized by dynamic light scattering (DLS) (Zetasizer Nano-S90, Malvern instruments, Malvern Panalytical, Malvern, UK) at a fixed 90° scattering angle at 25 °C. The sample was suspended in ultrapure water and the measurements were performed in triplicate.

### 2.6. Scanning Electron Microscopy (SEM)

The morphology and particle size of the NanoC and NanoS were evaluated by a field emission-scanning electron microscope (FE-SEM Zeiss Sigma 300, Zeiss, Germany). The SEM sample stage was prepared by placing double-sided adhesive carbon tape on an aluminum stub. One small drop of 1 mg/mL nanohydrogel sample suspended in ultrapure water was placed on the sample stage and then dried at 37 °C overnight. Subsequently, the dried sample was sputtered under vacuum with a chromium layer of approximately 100 Å thickness (Quorum Q150T ES, Quorum Technologies, Lewes, UK) prior to analysis.

### 2.7. Transmission Electron Microscopy (TEM)

The morphology and core-shell structure of NanoC were determined using transmission electron microscopy (TEM, Philips CM10, Philips Electron Optics, Eindhoven, The Netherlands) at an acceleration voltage of 80 kV and ranging from 92,000 to 130,000. The TEM sample stage was prepared using a copper grid. For detailed morphological analysis, specimens were processed for transmission electron microscopy observation using the conventional negative staining procedure. Eighty microliters of nanohydrogel were adsorbed onto formvar carbon-coated 200 mesh grids (Agar Scientific Ltd., Stansted, UK) for 10 min. Subsequently the grids were incubated with 2% *w*/*v* sodium phosphotungstate for 1 min and the excess liquid of the sample was removed by filter paper and the sample dried at room temperature.

### 2.8. Fourier Transform Infrared Spectroscopy (FT-IR)

Infrared spectra of the nanohydrogel and synthesized polymer were recorded by a Fourier transform infrared spectrophotometer (FT-IR, PerkinElmer, Milan, Italy) at a wavelength range of 4000–500 cm^−1^. All the spectra were recorded against a background of an air spectrum.

### 2.9. Raman Spectroscopy (RS)

The chemical structure of the NanoC, NanoS and synthesized polymer were characterized by Raman spectroscopy (RS). Raman spectra were recorded by a micro-Raman spectrometer (iH320, Horiba, Kyoto, Japan) in backscattering geometry and a microscope (Olympus BXFM-ILHS, Olympus Corporation, Tokyo, Japan).

A diode-pumped solid-state laser of 532 nm emission wavelength was used as the excitation source. Raman scattering light was collected using a 50× microscopy objective and dispersed with a 600 grooves mm^−1^ grating and detected using a cooled charge coupled device array detector (Horiba Syncerity, Horiba, Japan).

### 2.10. Redox Responsive Assay of the Blank Nanohydrogel

GSH was used as a reducing agent to study the redox responsiveness of the nanohydrogel. The NanoC and NanoS prepared by different cross-linking times at 24, 48 and 72 h were studied. In detail, 1 mg nanohydrogel was suspended in 1 mL PBS (10 mM, pH 7.4) or 1 mL PBS supplemented with 10 μM or 10 mM GSH to mimic the extracellular and intracellular environments, respectively. The nanohydrogel suspension was incubated at 37 °C at an optimized shaking speed of 100 rpm. With the aim of studying the redox-responsiveness of the nanohydrogel in the previously described buffered solutions, particle size was monitored at predetermined intervals by DLS. The particle size was considered as an indirect indicator of their degradation trigged by reducing environment.

### 2.11. Encapsulation of Protein in the NanoC and NanoS

Three model proteins CC, HRP and BSA differing in Mw (Mw_CC_ = 12.5 kDa, Mw_HRP_ = 44 kDa and Mw_BSA_ = 64 kDa) were loaded into the nanohydrogel to study the loading capability and release behavior of the developed NanoC and NanoS. Each protein was encapsulated by dissolving it in the water phase at a concentration of 10 mg/mL before the homogenization step of nanohydrogel preparation (Figure 1). Then, the protein containing water phase was added to the organic phase with or without Trib-sulf copolymer for the formulation of NanoC and NanoS. The nanohydrogel was prepared and purified following the previously described procedure at a homogenization speed of 13,500 rpm for 45 min, and then cross-linked under gentle stirring at 37 °C for 72 h. The protein loaded nanohydrogels were washed with ultra-pure water and precipitated using centrifugation (11,000 rpm, 30 min, 4 °C) three times to remove unencapsulated proteins, and finally isolated by lyophilization in the presence of 7% *w*/*v* sucrose as a lyoprotectant. To determine the encapsulated amount of protein, the protein loaded nanohydrogel was suspended in PBS (10 mM, pH 7.4) and incubated with the reagents of the Micro BCA assay for 2 h at 37 °C. Then, absorbance at 562 nm was detected using a microplate reader (FLUOstar^®^ Omega, BMG LABTECH, Ortenberg, Germany). The protein encapsulation efficiency (EE) and loading capacity (LC) were calculated according to the Equations (1) and (2).

(1) $$\mathrm{Encapsulation}\;\mathrm{efficiency}\;\left(\mathrm{EE},\;\%\right)=\frac{\mathrm{Weight}\;\mathrm{of}\;\mathrm{loaded}\;\mathrm{protein}}{\mathrm{Weight}\;\mathrm{of}\;\mathrm{feeded}\;\mathrm{protein}}\times 100\%$$

(2) $$\mathrm{Loading}\;\mathrm{capacity}\;\left(\mathrm{LC},\;\%\right)=\frac{\mathrm{Weight}\;\mathrm{of}\;\mathrm{loaded}\;\mathrm{protein}}{\mathrm{Weight}\;\mathrm{of}\;\mathrm{polymer}}\times 100\%$$

### 2.12. In Vitro Protein Release

The release of loaded protein from the NanoC and NanoS was investigated in two different release media: plain PBS buffer (10 mM, pH 7.4, 136 mM NaCl) or PBS buffer supplemented with 10 mM GSH to simulate the extracellular and intracellular environment, respectively. The protein loaded nanohydrogel was suspended into release media at a concentration of 5 mg/mL and then the nanohydrogel suspension was transferred into a dialysis device with a Mw cutoff at 100 kDa (Spectra-Por^®^ Float-A-Lyzer^®^ G2, Sigma-Aldrich, St. Louis, MO, USA) in triplicate. The dialysis device was submerged into a falcon test tube with14 mL release medium, incubated at 37 °C and gently shaken at 50 rpm. At desired time intervals, 0.6 mL of release medium was sampled and replenished with an equal amount of fresh medium. The released protein was determined by Micro BCA assay. The released sample of GSH-containing medium was further purified by dialysis (Mw cutoff 3.5 kDa) against 0.01 M pH 7.4 PBS for 3 days before the Micro BCA assay to remove GSH that interferes with the colorimetric assay.

### 2.13. Peroxidase-like Enzymatic Activity Assay of Encapsulated HRP and CC

The peroxidase-like enzymatic activities of free HRP and HRP loaded NanoC/NanoS were examined by 2,2′-azinobis-(2-ethylbenzthiazoline-6-sulfonate) (ABTS) [63]. An ABTS substrate solution was freshly dissolved in potassium phosphate buffer (100 mM, pH 5.0). This substrate displays strong UV absorption at 340 nm with a molar extinction coefficient of 3.66 × 10^4^ M^−1^cm^−1^. In the presence of an oxidizing agent, such as HRP, ABTS turned into ABTS^+^, that displays a UV shift its absorption maximum at 405 nm. Free HRP or HRP loaded NanoC/NanoS were diluted using potassium phosphate buffer (40 mM, pH 6.8) containing 0.23% *w*/*v* BSA and 0.5% *v*/*v* Triton X-100^2^. The assay mixture containing the final concentration of ABTS ranged from 10 to 8000 μM, H_2_O_2_ 3.99 mM and 0.08 μM HRP in a total volume of 245 μL. The increase in absorbance at 405 nm was monitored immediately after adding the H_2_O_2_ into the reaction mixture using a time-scan model of microplate reader every 20 s for 2 min.

The peroxidase-like enzymatic activity of free CC and CC loaded NanoC/NanoS was also measured by a colorimetric assay using ABTS/H_2_O_2_ according to the study of Kim et al. [64] with modifications. Fresh ABTS substrate solution was prepared by dissolving in PBS buffer (10 mM, pH 7.4). Free CC or CC loaded nanohydrogels were diluted using PBS (10 mM, pH 7.4). The assay mixture containing final concentration of ABTS ranged from 0.5 to 16 mM, H_2_O_2_ 16 mM and 0.9 μM CC in a total volume of 245 μL. The increase in absorbance at 415 nm was read immediately after adding H_2_O_2_ into the reaction mixture using a time-scan model of a microplate reader every 20 s per time for 2 min.

The peroxidase-like enzymatic kinetic parameters were calculated by the Michaelis-Menten equation:

(3) $$v=V_{max}\times \left[S\right]/\left(K_{m}+\left[S\right]\right)$$

*v*: the initial rate of the reaction

*V_max_*: maximum rate of the reaction

[*S*]: substrate concentration

*K_m_*: Michaelis constant

### 2.14. Cell Culture

A RAW 264.7 macrophage cell line (Sigma, St. Louis, MO, USA) was cultured in Dulbecco’s modified eagle medium (DMEM) supplemented with 10% fetal calf serum (FCS) and 1% penicillin-streptomycin (PS) in a cell incubator at 37 °C under a 5% CO_2_ atmosphere.

### 2.15. In Vitro Cytotoxicity Assay

The in vitro cytotoxicity assay was performed using an LDH cytotoxicity assay kit purchased from Thermo Fisher Scientific (Waltham, MA, USA). The reagents used in the following procedure including Lysis Buffer (10X), Reaction Mixture and Stop Solution were provided originally from the kit. RAW 264.7 cells were seeded in 96-well plates at a density of 1 × 10^4^ cells per well. The cells were cultured in 100 μL DMEM at 37 °C and 5% CO_2_ in an incubator overnight for cell attachment. Then, the medium was replaced by the NanoC medium suspension. The cells were co-incubated with NanoC medium suspension for 24 h. NanoC suspensions were prepared in culture medium at concentrations of 50, 100, 500, 1000, 1500, 2000 and 3000 μg/mL. Two groups of cells were cultured without exposure to NanoC as spontaneous LDH activity controls and maximum LDH activity controls. The medium was harvested after 24 h. Forty-five mins before harvesting the medium, 10 μL of Lysis Buffer (10X) was added in to target maximum LDH activity controls. Fifty microliters of each sample medium were transferred to a 96-well plate and 50 μL of reaction mixture was added to each well and gently mixed. The plate was protected from light and incubated at room temperature for 30 min. Subsequently, the reaction was stopped by adding 50 μL of Stop Solution to each well. To determine LDH activity, the 680 nm absorbance value (background) was subtracted from the 490 nm absorbance value (SpectraMax iD3, Molecular Devices, San Jose, CA, USA) and the cytotoxicity percentage was calculated by Equation (4).

(4) $$\%\mathrm{Cytotoxicity}=\frac{\mathrm{Compound}-\mathrm{treated}\;\mathrm{LDH}\;\mathrm{activity}-\mathrm{Spontaneous}\;\mathrm{LDH}\;\mathrm{activity}}{\mathrm{Maximum}\;\mathrm{LDH}\;\mathrm{activity}-\mathrm{Spontaneous}\;\mathrm{LDH}\;\mathrm{activity}}\times 100\%$$

### 2.16. Confocal Laser Scanning Microscopy (CLSM)

Hydrophilic dye fluorescein isothiocyanate (FITC) as a fluorescent tracker was encapsulated into NanoC by dissolving into water phase at a concentration of 0.1 mg/mL during NanoC formulation. Internalization of FITC loaded NanoC by RAW264.7 macrophage cells was confirmed by confocal laser scanning microscopy (CLSM, Leica SP5 CLSM, Leica, Germany). RAW264.7 macrophage cells were seeded on coverslips in a sterile 6-well plate at a density of 2 × 10^5^ cells per well and incubated in DMEM complete medium overnight. Then, the cells were washed three times by PBS and treated with 1 mL NanoC DMEM complete medium solution (1 mg/mL) per well. Cells were co-incubated with NanoC at 37 °C for 6 h. The supernatant was removed and the cells were washed three times with PBS. Subsequently, lysosome, cell membrane and nuclei were stained by different fluorescent dyes. LysoTracker^TM^ Deep Red was added at the concentration of 60 nM and incubated for 1 h at 37 °C to visualize the lysosomes of cells. Cells were then washed with PBS three times to remove the excess dye and fixed by 4% paraformaldehyde solution for 15 min. Later, cell membrane and nuclei were dyed by PKH 26 Red fluorescent cell linker and DAPI, respectively. The coverslip with fixed and dyed cells were sealed on the object slide and imaged by confocal microcopy. Nuclei, NanoC, cell membrane and lysosome were observed by respective channels at 405 nm (blue), 488 nm (green), 561 nm (red) and 633 nm (deep red).

## 3. Results and Discussion

### 3.1. Characterizations of Synthesized Trib-Sulf and HA-SH Polymers

Vinyl sulfone groups were introduced to act as cross-linkable moieties for the combination between Trib-sulf and HA-SH during NanoC formulation. The main characteristics of the Trib-sulf copolymer before and after the vinyl sulfonation are summarized in Table S1. The average molecular weight (Mn) of the synthesized Trib-sulf was 37 kDa by gel permeation chromatography (GPC). The chemical structure of Trib-sulf was determined by proton nuclear magnetic resonance (^1^H-NMR) and the spectrum displayed in Figure S3. The substitution degree (DS%) of vinyl sulfone groups calculated based on ^1^H-NMR spectrum and Equation (S1) was 12%, a value close to the aimed DS% of 10%. The LCST of Trib-sulf was 31 °C.

HA-SH was synthesized with a quantitative yield of 99.80%. The chemical structure and thiolated DS% were measured by ^1^H-NMR (Figure S4). The thiolated DS% was around 27% based on Equation (S2).

More details and discussions of Trib-sulf and HA-SH synthesis and characterization can be found in the Supplementary Data.

### 3.2. Factor Influence Analysis

**Figure 2:**
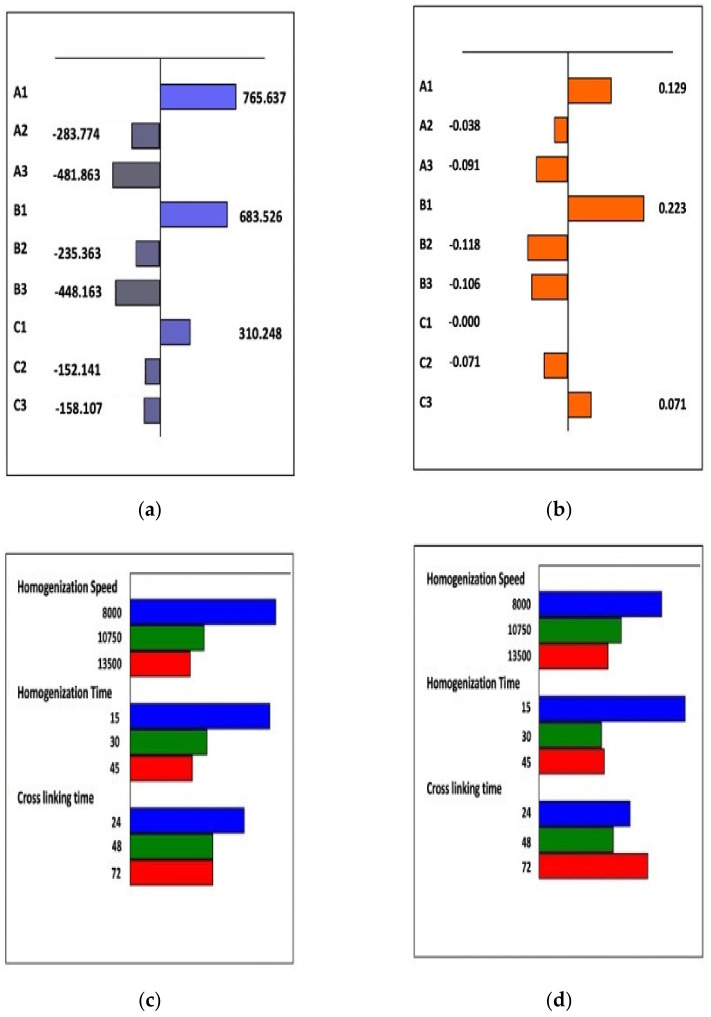
Graph mode representation of partial and total effects associated with the factor levels for: (**a**,**c**) the nanoparticle size; (**b**,**d**) polydispersity index (PDI) of NanoC. Effect estimations were obtained after NEMROD analysis by using the least square methods. The factor effect levels are expressed in coded values as reported in Table 2.

Having verified the feasibility of the experimental plan (Table 2), 27 trials were randomly run to reduce the effect of external (uncontrolled) factors. The effects of each factor were estimated by means of the least square method using NEMRODW^®^ software on the basis of the results obtained. The variation of the experimental response was graphically analyzed to identify the factor level providing the minimum response variable (Figure 2).

The effect diagrams shown in Figure 2a,c allowed us to identify the factor levels that most significantly contributed to obtaining nanoparticle size of about 200–300 nm. These factor levels are the levels A3, corresponding to the homogenization speed of 13,500 rpm, and B3 corresponding to the homogenization time of 45 min, whereas for the cross-linking time it turned out that there was no difference in the contribution of C2 and C3 levels. With regard to the PDI, on the basis of the effect diagrams in Figure 2b,d, it was possible to see an influence of the homogenization speed levels similar to those determined for the nanoparticle size with a preference, also in this case, for the A3 level. For the homogenization and cross-linking times, with regard to the PDI, B2 and B3 did not affect the homogenization time, while the C2 level was preferred for the cross-linking time. Analysis of half-normal and normal plots (not reported here) also confirmed the same activity for the factor levels graphically represented above, without evidence of active interactions among the different factor levels here considered.

### 3.3. Characterization of the NanoC Prepared by the Selected Parameter Conditions

The selected conditions for the NanoC preparation, in terms of particle size of approximately 250 nm and narrow size distribution, were identified by design of experiments (DOE) with a homogenization speed of 13,500 rpm and homogenization time of 45 min. No significant difference in particle size prepared in these conditions was observed when different cross-linking times of 24, 48 and 72 h were applied, explained by the fact that the highest homogenization time and speed formed a nanoemulsion consisting of smaller nanodrops. However, it is supposed that the cross-linking time may cause differences in shell thickness, which did not affect the overall particle size but had the potential to further affect the degradation and drug release behavior of the NanoC. Therefore, three batches of NanoC using the optimized homogenization speed and time (13,500 rpm, 45 min) with different cross-linking times of 24, 48 and 72 h were prepared and characterized for further applications as a delivery system. Additionally, to study the influence of the core-shell structure on the cargo delivery behavior, three batches of nanohydrogel without shell, i.e., NanoS, were prepared in the same conditions of the NanoCs. The preparation conditions and abbreviations of each batch are summarized in Table 3.

**Figure 3:**
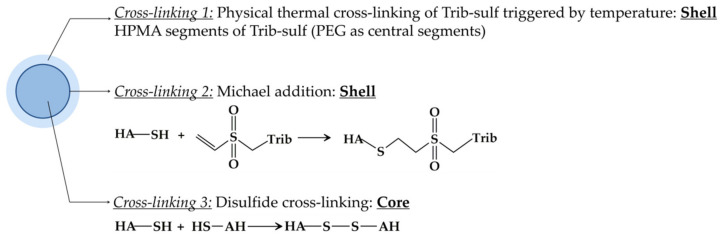
Schematic representation of the novel HA-SH/Trib-sulf NanoC designed in this study.

The NanoC was formulated by the W/O nanoemulsion method, and cross-linking occurred as a consequence of three simultaneous mechanisms (Figure 3). This interfacial Michael addition cross-linking reaction was possible due to the different solubility of the HA-SH and Trib-sulf copolymer in water and CHCl_3_, used as immiscible solvents for the preparation of the nanoemulsion. As a derivative of HA, HA-SH has high solubility in water whilst it is insoluble in most organic solvents, including CHCl_3_, whereas the Trib-sulf copolymer is soluble in both CHCl_3_ and water at the temperature lower than LCST (31 °C). Thus, the HA-SH aqueous solution was added into CHCl_3_ dropwise and cut into nanoscale drops by homogenization, thereby forming a nanoemulsion. Simultaneously, due to moderate solubility in water, the Trib-sulf copolymer dissolved in the CHCl_3_ organic phase started to diffuse towards the water drop and reach the water/organic interface [58]. During the incubation of the nanoemulsion at 37 °C, the permeated Trib-sulf into the water phase underwent thermal gelation owing to the thermosensitive poly-HPMAm segments. Then, an interfacial Michael addition reaction started between the thiol groups of HA-SH and vinyl sulfone groups of Trib-sulf along the interphase of the nanodrops [65]. The covalent cross-linking can efficiently stabilize the shell structure of the nanohydrogel [66]. In pharmaceutical and biomedical applications [67,68,69], the Michael addition reaction, which efficiently favors the coupling of electron poor olefins with nucleophiles [70], offers several advantages over other covalent crosslinking methods. The Michael addition can spontaneously occur under physiological conditions without using any toxic catalysts. Meanwhile, disulfide bonds were formed via the oxidation process of thiol groups of HA-SH inside the core of the nanodrops. The stable biphasic dispersion of the HA-SH water phase in the Trib-sulf organic phase was stabilized by lecithin as a surfactant.

The particle size, PDI and zeta potential of the obtained NanoC and NanoS were measured by DLS and are summarized in Table 3. Particle size was demonstrated to play a significant role in influencing the efficiency and pathway of nanoparticle cellular uptake. It is proved that nanoparticles of particle size in the range of 5–250 nm have the ability to overcome biological barriers, thereby increasing the effect of targeted therapy [71,72]. All the developed NanoC and NanoS presented an average diameter around 250 nm with the potential to be developed as ideal systemic drug delivery systems. Considering that there was no significant difference in particle size between NanoC and NanoS, it can be hypothesized that the shell thickness of the nanocapsule generally formed by nanoemulsion method falls in the range of a few nanometers [66] so that DLS measurement may not be sufficiently sensitive to detect the difference in particle size between NanoC and NanoS. Additionally, compared with NanoS, the three batches of NanoC showed lower PDI. This observation can be attributed to the presence of Trib-sulf in the formulation of NanoC. The hydrophilic central PEG segment and the hydrophobic poly-HPMAm-lactated_1-2_ side groups give Trib-sulf an amphipathic nature. Therefore, beside lecithin, Trib-sulf acted as an additional surfactant for the nanoemulsion preparation of NanoC, which increased the stability of the nanoemulsion, enabling the final NanoC to have a lower size distribution compared with NanoS. Concerning the zeta potential, all the nanohydrogels displayed a negative value, which is consistent with the anionic nature of HA owing to the abundant carboxylic groups. However, compared with the natural zeta potential of HA (~−40 mV [73]), the zeta potential of NanoC and NanoS were both increased, reaching average zeta potentials around −15 mV and −25 mV, respectively. For the NanoS, it can be explained that 30% of the carboxylic groups were substituted by thiol groups of HA-SH polymer, thereby the negative charges of HA were partly shielded. On this basis, the HA core of NanoC was further covered by Trib-sulf copolymer which possessed electric neutrality. Therefore, NanoC presented a zeta potential closer to neutrality compared with NanoS.

**Figure 4:**
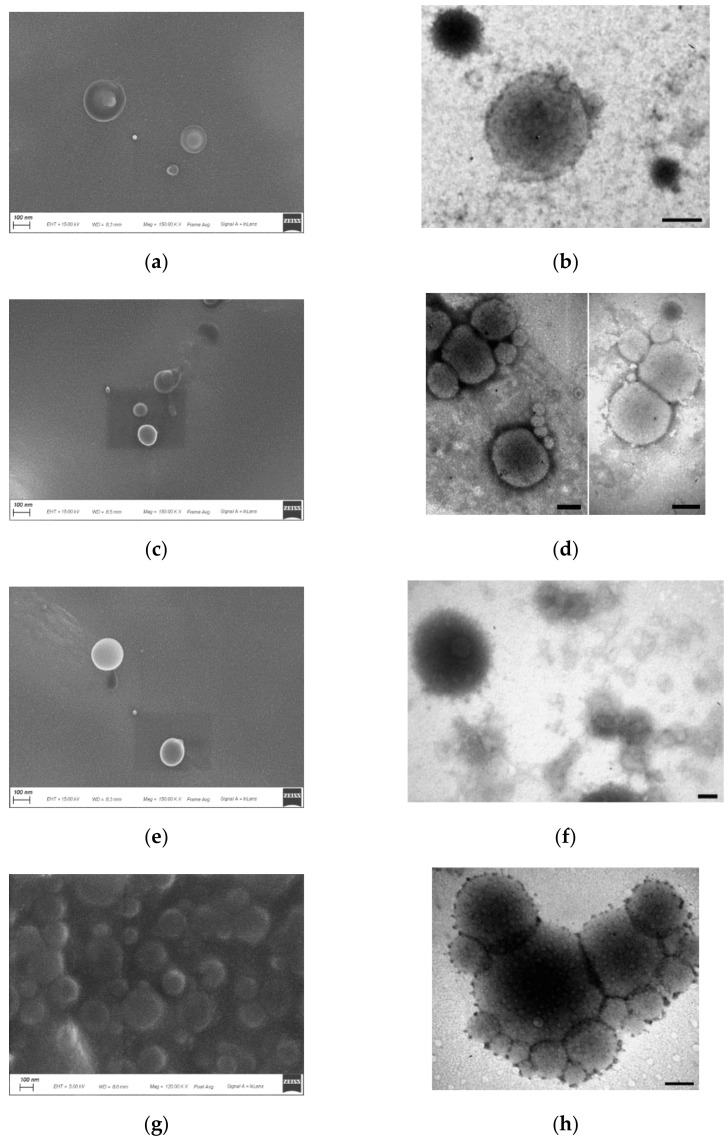
SEM and TEM imaging of NanoC cross-linked by different times: (**a**) SEM of NanoC_24; (**b**) TEM of NanoC_24; (**c**) SEM of NanoC_48; (**d**) TEM of NanoC_48; (**e**,**g**) SEM of NanoC_72; (**f**,**h**) TEM of NanoC_72 (**b**,**d**,**f**,**h** scale bar = 100 nm).

**Figure 5:**
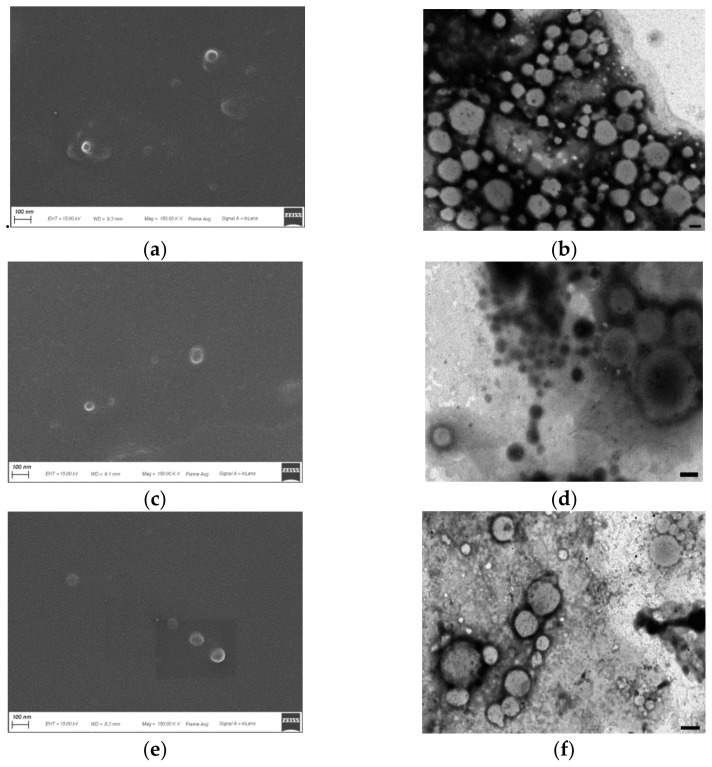
SEM and TEM imaging of NanoS cross-linked by different times: (**a**) SEM of NanoS_24; (**b**) TEM of NanoS_24; (**c**) SEM of NanoS_48; (**d**) TEM of NanoS_48; (**e**) SEM of NanoS_72; (**f**) TEM of NanoS_72 (**b**,**d**,**f** scale bar = 100 nm).

To describe morphology and core-shell structure, all batches were characterized by SEM and TEM (Figure 4 and Figure 5). For a detailed morphological analysis by TEM, vesicles were processed for negative staining, a technique that allows highlighting of the cell membranes by coloring the outside, if the membrane is intact, and leaving the core in a lighter color [74,75]. As shown in SEM images, all the nanohydrogels adopted a regular spherical shape and smooth surface, which confirmed that both NanoS and NanoC were well cross-linked. The particle size of all the nanohydrogels coarsely estimated by SEM and TEM were smaller than those obtained by DLS. In detail, all the NanoC cross-linked by different times (from 24, 48 to 72 h) presented a uniform particle size of around 100–150 nm by SEM, which is smaller than the value detected by DLS of around 250 nm. This result can be explained as follows. The nanohydrogel sample subjected to the DLS measurement was suspended in water and thus in its hydrated state, while samples to be observed by SEM or TEM were previously dehydrated [76]. Additionally, the two techniques (DLS from one side, and SEM or TEM from the other one) are based on different principles. DLS as an intensity based technique has higher emphasis on the particle with larger size, while the SEM/TEM imaging techniques measure the particle size by direct observation [77,78].

Compared with NanoC, the NanoS sample exhibited stronger size shrinkage from the hydrated state under the DLS to the dehydrated state under the SEM/TEM. It demonstrated that the hydrophobic HPMAm segment containing the Trib-sulf shell provided a better mechanical support during the dehydration process of SEM/TEM sample preparation. The core-shell structure was confirmed under the TEM. A membrane structure composed of coherent small vesicles around the nanohydrogel was observed in all the three batches of NanoC. A discontinuous membrane stain can be seen in the TEM image of NanoC_24. When the cross-linking time increased to 48 h, a more continuous vesicle membrane was observed. Interestingly, the NanoC_72 presented a well-defined membrane structure compared with the other NanoC_24 and NanoC_48 which may suggest that thickness of the NanoC may increase with cross-linking time. In contrast, the matrix structure nanohydrogel presented only a transparent core under TEM as sufficient evidence for the existence of a shell for the core-shell structure nanohydrogels.

### 3.4. Characterization of Chemical Cross-Linking of the NanoC and NanoS

Considering the direct observation of the shell structure of NanoC by TEM imaging, we were interested in confirming the actual interfacial crosslinking of the NanoC by chemical characterization. NanoC_72 and NanoS_72 were characterized by FT-IR and RS using HA-SH and Trib-sulf copolymer as references.

**Figure 6:**
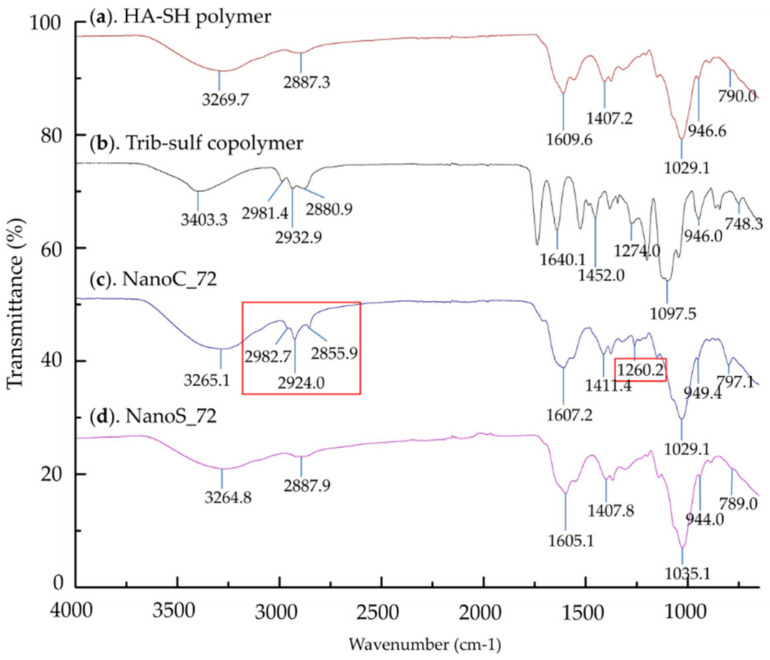
FT-IR spectra of (**a**). HA-SH polymer; (**b**). Trib-sulf copolymer; (**c**). NanoC_72 and (**d**). NanoS_72.

The FT-IR spectra of NanoC_72 and NanoS_72 depicted in Figure 6 were compared with those of HA-SH and Trib-sulf copolymer, considered as control samples. It is possible to confirm the presence of HA-SH in both NanoC_72 and NanoS_72 nanohydrogels when comparing the spectrum with the HA-SH polymer. In detail, the presence of the characteristic peaks of HA presented in the spectrum of HA-SH and nanohydrogel is explained as follow. The peak at around 3260 cm^−1^ in the spectrum of HA-SH polymer (3269.7 cm^−1^), NanoC_72 (3265.1 cm^−1^) and NanoS_72 (3264.8 cm^−1^) corresponds to the stretching of NH and OH groups of HA [79,80,81]. The peaks at around 1600 cm^−1^ (1609.6, 1607.2 and 1605.1 cm^−1^ in the spectrum of HA-SH polymer, NanoC_72 and NanoS_72) and 1400 cm^−1^ (1407.2, 1411.4 and 1407.8 cm^−1^ in the spectrum of HA-SH polymer, NanoC_72 and NanoS_72) are attributed to the vibration of amide carbonyl and stretching of carboxyl bonds corresponding to the acid group of HA [79,80,81]. Additionally, the absorption peak at around 1029 cm^−1^ and 946 cm^−1^ corresponds to the stretching of –C–O–C– in the main chain of HA [79,80,81]. It can be noted that in the spectrum of NanoC_72 (Figure 6c), besides the typical characteristic peaks of HA, there were three new peaks at 2982.7, 2924.0 and 2855.9 cm^−1^ also present in the spectrum of the Trib-sulf copolymer that corresponded to the stretching vibration of the alkanes. Another new peak at 1260.2 cm^−1^ is attributed to the stretching of –C–O– in the PEG segment of Trib-sulf copolymer [82]. On the other side, the spectrum of NanoS_72 (Figure 6d) presented substantial agreement with the HA-SH polymer without discovery of the new peak, which proved that NanoS was composed of only one chemical component, HA-SH. These results confirmed that the NanoC consisted of a high proportion of HA-SH polymer and a relative low proportion of the Trib-sulf copolymer. However, the absorption peak of significant groups, including the thiol group, the vinyl sulfone group, the disulfide bond, and carbon-sulfur bond, which are supposed to form during interfacial crosslinking, were not detected by FT-IR due to their weak absorptive intensity of infrared radiation [83].

**Figure 7:**
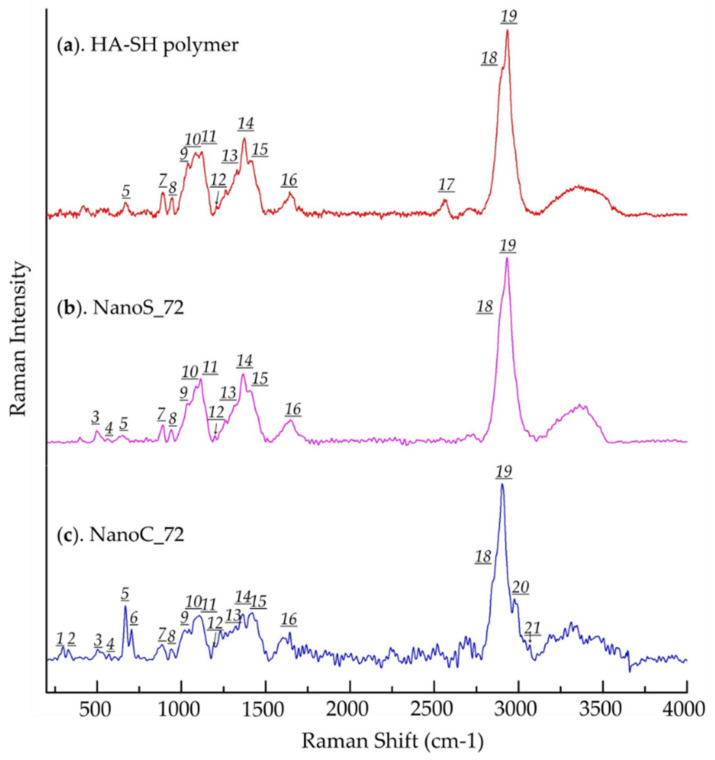
Raman spectra (RS) of (**a**). HA-SH polymer; (**b**). NanoS_72 and (**c**). NanoC_72.

To further confirm that the Michael addition reaction occurred on the shell, with the disulfide cross-linking in the core, NanoC_72 and NanoS_72 were further characterized by RS. HA-SH and the Trib-sulf copolymer were examined as references. The Raman spectra of HA-SH polymer, NanoS_72 and NanoC_72 are presented in Figure 7 and Table 4 summarizing the assignment of Raman bands observed for HA-SH, nanohydrogels and the corresponding data cited from the literature. The Raman spectrum of HA-SH polymer exhibited all the characteristic peaks of HA [81,84,85] with two specific peaks at 2656 and 665 cm^−1^ assigned to the –S–H bond and –C–S– linkage between the thiol group and the HA main chain which referred to the thiol group of the HA-SH polymer [86]. Similar to the FT-IR results, the Raman spectra of both NanoS_72 and NanoC_72 maintained most of the characteristic peaks of HA, evidencing that HA represented the majority of both NanoC and NanoS. Indeed, the comparison between the spectra of HA-SH and NanoC_72 revealed the disappearance of the thiol group peak at 2656 cm^−1^, while the appearance of two new peaks at 496 and 561 cm^−1^ corresponded to the stretching and bending vibration of disulfide bonds. This result indicated that the thiol groups of the HA-SH polymer were consumed and that the nanohydrogels were chemically cross-linked by disulfide bonds derived from the thiol groups of the HA-SH polymer. The same result was detected in NanoC_72, and two new peaks at 505 and 569 cm^−1^ demonstrated the existence of disulfide bonds. Additionally, the outcome of the Michael addition between thiol group of HA-SH to the vinyl sulfone group of Trib-sulf was confirmed by the presence of new peaks at 297 and 332 cm^−1^, that can be assigned to the bending vibration of the –SO_2_ bond of vinyl sulfone group, and the peak at 703 cm^−1^ attributed to newly formed –C–S– bonds between thiol and vinyl sulfone groups. Moreover, the appearance of the new peak at 2975 cm^−1^ assigned to the PEG segment of Trib-sulf also showed cross-linking between HA-SH and Trib-sulf.

As a conclusion to this subsection, the results of FT-IR spectroscopy and Raman spectroscopy showed complementarity. The FT-IR spectrum evidenced the NanoC composed by Trib-sulf and HA-SH polymers, while the Raman results proved that these two components were successfully chemical cross-linked with each other, i.e., Michael addition reaction between HA-SH and Trib-sulf, and self-linked by disulfide bonds among HA-SH chains. Thus, the results of FT-IR and Raman spectroscopy confirmed the core-shell structure of the NanoC from a chemical point of view.

### 3.5. Redox Responsive Degradation of NanoC and NanoS

**Figure 8:**
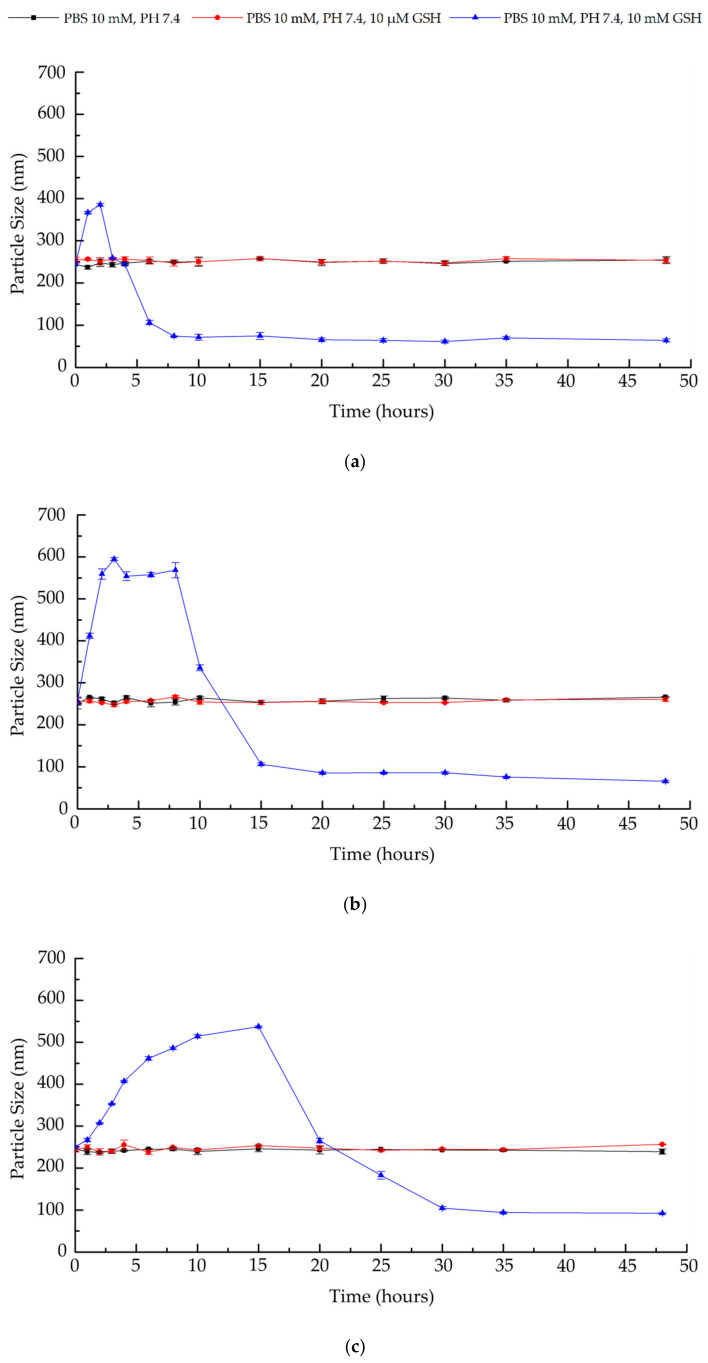

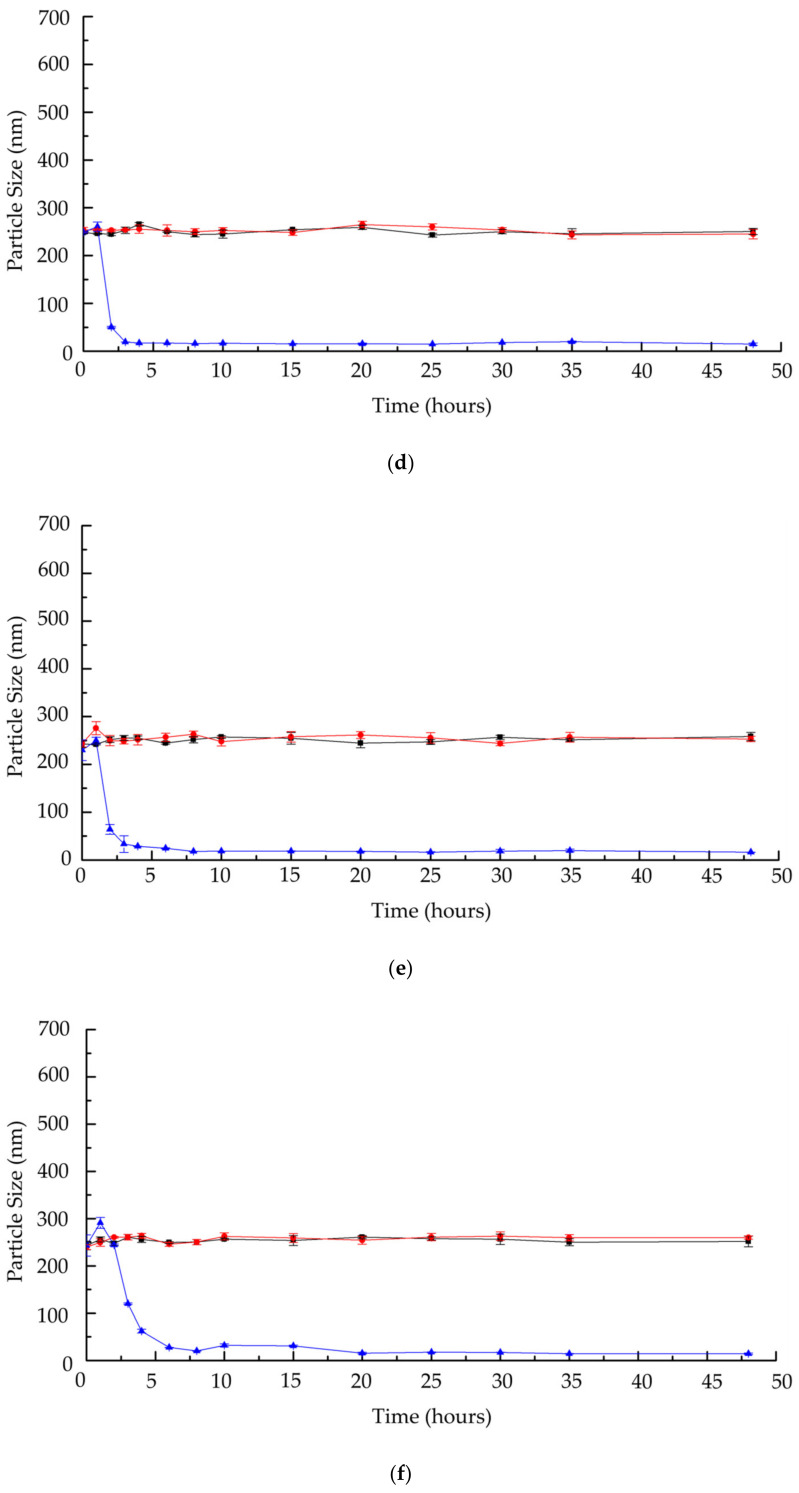
Characterization of the nanohydrogel redox responsiveness by particle size measurements using DLS. The measurements were performed in triplicate. (**a**) NanoC_24; (**b**) NanoC_48; (**c**) NanoC_72; (**d**) NanoS_24; (**e**) NanoS_48 and (**f**) NanoS_72.

NanoC was characterized by HA-S-S-AH disulfide linkages and HA-SH/Trib-sulf Michael addition double chemical crosslinking. Disulfide linkages are stable at physiological conditions, while they can be rapidly reduced to free thiol groups, leading to nanohydrogel degradation in a reducing environment, such as the intracellular or tumor environment [92,93]. On the other hand, the carbon-sulfur bond formed between HA-SH and Trib-sulf via the Michael addition is stable in both the physiological condition and reducing environment [94]. To further understand the redox responsiveness of the nanohydrogel and the position of disulfide linkages, particle size of the nanohydrogel was monitored by DLS along with time to demonstrate the degradation behavior of the nanohydrogel. Different amounts of GSH were added to the nanohydrogel PBS (10 mM, pH 7.4) suspension to achieve final GSH concentrations of 10 μM and 10 mM in order to mimic the extracellular and intracellular environments, respectively [95]. The sample in the absence of GSH was measured as a negative control. As described in Figure 8a–f, all the NanoC and NanoS samples prepared at different cross-linking times were stable in PBS without, or supplemented with, 10 μM GSH, as demonstrated by their particle size stability as a function of time. This observation indicated that both NanoS and NanoC can retain their structure and protect the payload in extracellular-mimicking conditions. Conversely, in the PBS containing 10 mM GSH, the three batches of NanoC differing in cross-linking time, showed an initial increase in particle size in the first 2–15 h, corresponding to nanohydrogel swelling, followed by particle size reduction owing to progressive network degradation. The NanoC formulated by the shorter cross-linking time of 24 h (Figure 8a) displayed a more moderate swelling, followed by faster degradation compared to similar formulations of longer crosslinking (48 and 72 h), where the swelling ratio and the swelling time positively correlated with the crosslinking time (Figure 8b,c). The shell of NanoC showed the capacity to resist the swelling stress and improve the stability of the nanohydrogel from 2 to 15 h depending on the cross-linking time. Eventually, the excessive internal pressure, due to the swelling of the core, resulted in shell disruption and nanohydrogel collapse. Different from NanoC, all NanoS systems showed similar behavior (Figure 8d–f), with a sharp decrease in particle size immediately after exposure to GSH-rich medium, indicating a fast reduction of disulfide bonds and rapid nanosphere degradation in the first 2 h. Overall, these results confirm that the NanoC carriers actually possess a Trib-Sulf shell interfacially cross-linked to the HA core, that these chemical crosslinks are stable in reducing environment and that the crosslinking time affects the thickness of the shell, and thereby nanosphere stability [50,96,97]. The presence of the outer shell in NanoC, besides increasing nanoparticles stability, can also play a role in controlling the release rate of the cargo, which could depend on core network degradation and diffusion through the shell pores.

### 3.6. Encapsulation of Protein into the NanoC and NanoS

To investigate the encapsulation capacity of NanoC, three proteins, CC, HRP and BSA, differing in Mw (Mw_cc_ = 12.5 kDa, Mw_HRP_ = 44 kDa and Mw_BSA_ = 64 kDa) were used as therapeutic models, dissolved in the HA-SH water phase, and loaded into the nanohydrogel. According to the results of the redox-responsive degradation assay, a 72 h crosslinking time was used for protein encapsulation to obtain stable nanoparticles, to better prevent the premature leakage of drugs from the NanoC and possibly have enhanced control over release rates. Therefore, NanoC_72 and NanoS_72 were studied to verify how the Michael addition cross-linked Trib-sulf shell influenced the loading capacity and payload release. As overviewed in Table 5, the particle size of the protein-loaded nanohydrogel increased compared with the blank to a different extent. The surface zeta potential of the protein-loaded nanohydrogel was consistent with the blank, which indicated that the protein was entrapped in the hydrophilic core without any interaction on the surface of the nanohydrogel.

Interestingly, a high encapsulation yield was achieved even for a protein carrying the same charge as the nanohydrogel. For example, the NanoC or NanoS with a zeta potential at around −12 and −25 mV can effectively encapsulate BSA, which possesses a negative charge at pH 7.4, with an encapsulation yield of 93.41 ± 5.49% and 93.28 ± 6.59%, respectively. This result can be explained by the very low solubility of encapsulated protein in the chloroform which was used as the continuous phase of the nanoemulsion during the nanohydrogel preparation. Most of the protein stayed in the water core instead of diffusing into chloroform due to its high water solubility and insolubility in chloroform. Additionally, it was demonstrated that a kind of slowly dissociable bond can be formed between HA and some proteins, especially BSA, thereby promoting the retention of BSA inside the HA core of the nanohydrogel [98,99]. Considering that most cancer immunotherapeutics are peptide or protein-based and insoluble in chloroform, the water in chloroform nanoemulsion method can be considered a promising strategy of nanoparticle preparation for a macromolecule delivery system in cancer immunotherapy applications.

Additionally, a significant difference of encapsulation yield was obtained between the two metalloenzymes HRP and CC, which was around 50% and 90% respectively, for both NanoC and NanoS. The high encapsulation yield of CC may be due to the excellent solubility of CC in water and insolubility in chloroform that maximized its concentration in the hydrophilic nanodrops during the nanoemulsion cross-linking procedure. On the contrary, a partial phase separation phenomenon was observed for the nanoemulsion during the loading of HRP into NanoC or NanoS. Metalloenzymes are composed of a peptide segment with a different molecular weight and an iron heme group. For CC, the heme prosthetic group is covalently attached on the side chain of peptide, thereby forming an amphiphilic lipid-like chain with a heme head and a peptide tail [100]. This amphiphilic structure contributed to the stability of the nanoemulsion. On the contrary, the iron heme group of HRP is located at the center of peptide chain reducing its amphiphilic nature and has a molecular weight four times higher than CC, which may decrease the stability of the nanoemulsion, and consequently bring down the encapsulation yield of HRP [101].

### 3.7. Peroxidase-like Enzymatic Kinetics of HRP and CC Loaded NanoC and NanoS

**Figure 9:**
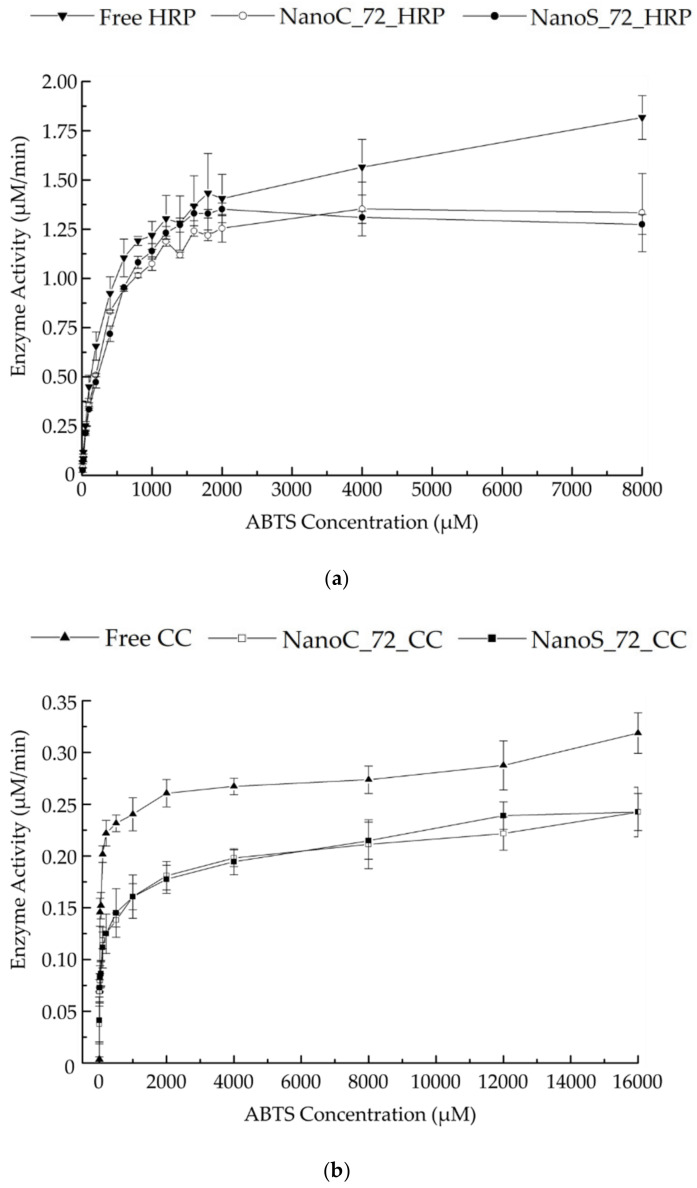
Peroxidase-like enzymatic activity of (**a**) free HRP, NanoC_HRP and NanoS_HRP; (**b**) free CC, NanoC_CC and NanoS_CC. The measurements were performed in triplicate.

To evaluate the influence of the encapsulation procedure on activity of the cargo, the peroxidase enzymatic activity of HRP and CC loaded nanohydrogels were determined. As shown in Figure 9 and Table 6, the nonlinear regression enzymatic kinetics of all the enzyme loaded nanohydrogel displayed good correlation (*R*^2^ > 0.99) with Michaelis-Menten kinetics. When compared the Michaelis constants (*K_m_*), transformation efficiencies (*k_cat_*) and maximum reaction rate (*v_max_*) with free HRP, the HRP-loaded NanoC showed a slight decrease around 11% of *k_cat_* and *v_max_* along with a 13% increase of *K_m_*. This may be caused by the additional light transmission resistance from the nanohydrogel network and also the spatial hindrance of the enzyme active site [102]. Nevertheless, the possibility to induce inactivation of the enzyme during the encapsulation procedure should not be neglected. While a higher increase of *K_m_* was observed of HRP-loaded NanoS compared with HRP-loaded NanoC, this result may imply that the Trib-sulf polymer can form a hydrophobic shell immediately once the nanoemulsion was transferred to 37 °C. Therefore, the encapsulated enzyme was better protected during the cross-linking procedure compared with NanoS. Similar results were found with CC loaded-NanoC and NanoS. It can be concluded that most of the peroxidase-like enzymatic activity was efficiently maintained during the nanohydrogel preparation procedure. Therefore, this NanoC can be considered as a promising delivery system for bioactive macromolecules.

### 3.8. In Vitro Controlled Protein Release and Kinetic Analysis

**Figure 10:**
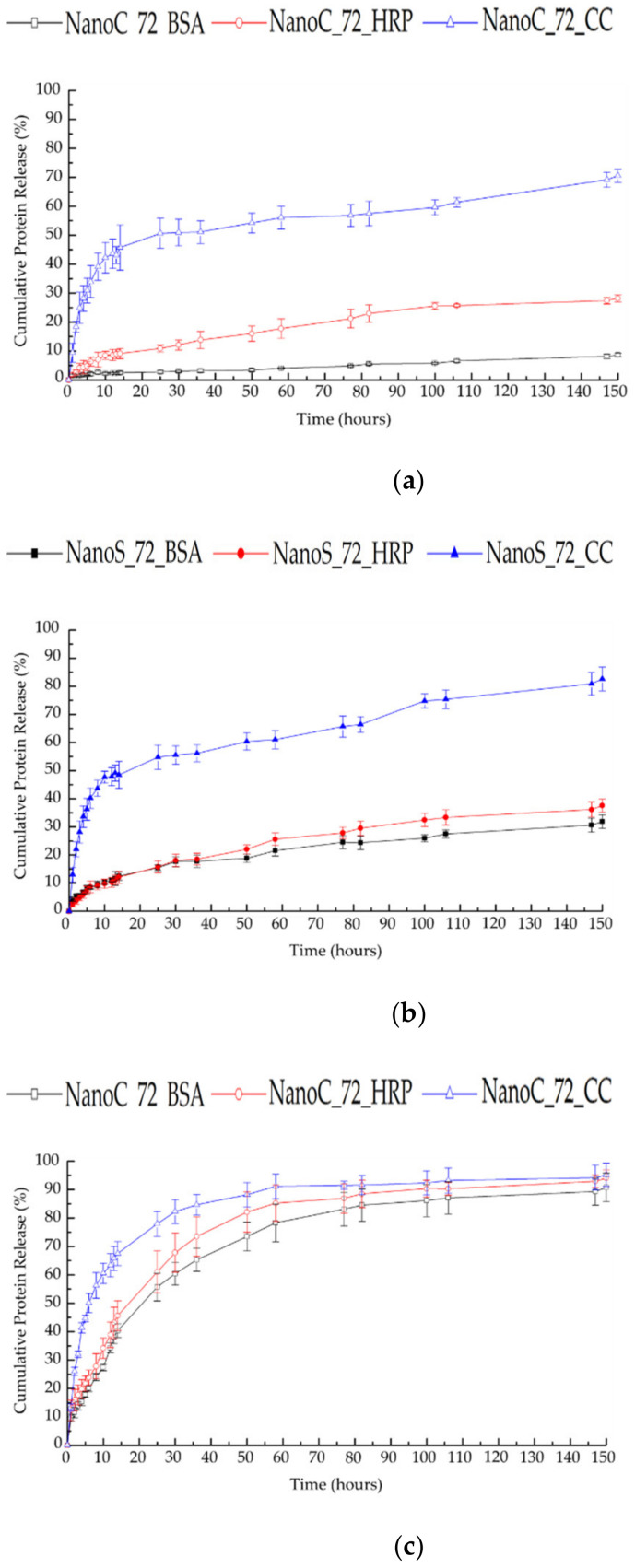

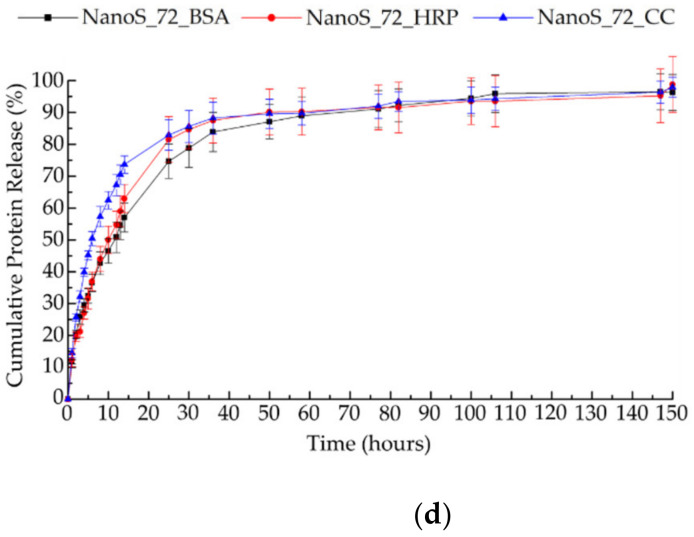
In vitro cumulative protein release of (**a**) NanoC_72 in PBS; (**b**) NanoS_72 in PBS; (**c**) NanoC_72, PBS with 10 mM GSH; (**d**) NanoS_72, PBS with 10 mM GSH. The measurements were performed in triplicate.

The cumulative release of proteins from NanoC_72 and NanoS_72 was studied in the different media with or without GSH to evaluate the redox responsive release behavior of the nanohydrogel and verify a possible correlation with the degradation profiles, as previously described (Figure 10). As shown in Figure 10a,b, all three different proteins were only partly released from both NanoC_72 and NanoS_72 in the physiological medium The release rates of three selected proteins from the NanoC_72 and NanoS_72 were clearly inversely correlated with the molecular weight of the selected proteins (release rate: CC > HRP > BSA) in the PBS medium. Compared with NanoS_72, all three proteins, especially BSA, showed a lower release rate of NanoC_72, which confirmed that the hydrophobic shell can effectively reduce leaking of the hydrophilic payload from the hydrophilic core. Additionally, the specific molecular interaction between HA and BSA can also promote the retention of BSA in the HA core of NanoC [98,99]. On the other hand, in the presence of 10 mM GSH, an initially fast release of all three proteins was observed in both NanoC_72 and NanoS_72 (Figure 10c,d) in the first 14 h, with an almost complete release achieved after 60 h. This result can be explained by the degradation behavior of the nanohydrogel. Disulfide bonds of the core can be cleaved in the reducing condition rapidly, thereby causing the fast release of protein. When comparing the slopes of the cumulative release, the NanoC_72 displayed slightly lower release rate than the NanoS_72 in the first 14 h because the Michael addition cross-linked hydrophobic shell was stable in the reducing environment, which can partly slow down the protein release from the degraded core.

To better explain the mechanism and kinetics of release, the release data were fitted into a kinetic model. The release kinetics of the proteins from the NanoC and NanoS at both the physiological medium and the reducing environment were investigated using zero-order, first-order and Ritger-Peppas kinetic models (see Supplementary Data) in order to gain more insight into the release mechanism [1,2,3]. The parameters and R^2^ according to different kinetic models for protein release from NanoC and NanoS are presented in Tables S2 and S3.

The analyzed parameter and R^2^ (Table S2) proved that, with the exception of NanoC_72_BSA, all the other samples followed a first-order release in the physiological medium according to the Ritger–Peppas model (*n* ≤ 0.5). The protein release for both NanoC and NanoS in PBS followed a Fickian-diffusion controlled release behavior [103]. An exception was observed for NanoC_72_BSA, where a zero-order release fitted the curve very well. This exception can be explained by the fact that soluble BSA cannot freely diffuse and is more restricted in the polymer network because the pore size of the NanoC is lower that the hydrodynamic radius of the BSA, so that the diffusion of the BSA from NanoC is slower.

Concerning the NanoC and NanoS in 10 mM GSH, fitting the curves with a first order or Ritger-Peppas kinetic models failed because the nanohydrogel is not stable with time and undergoes swelling and degradation as shown in Figure 8. This is why we proposed to identify the point where the nanohydrogel undergoes a structural modification with a consequent change in release mechanism and behaviors. Actually, it is possible to observe a rapid release for all the proteins during the first 14 h, corresponding to the degradation of the NanoC (Figure 8c), that was fitted with a zero-order release (Table S3). This result confirmed that the nanohydrogel was rapidly degraded due to the cleavage of disulfide bonds in the condition rich in GSH, thereby inducing a zero-order release profile by diffusion independently from the protein concentration. For the second part of the curve, which is the release behavior between 14 h and 150 h, all the samples exhibited a first-order release behavior dominated by a diffusion-controlled release mechanism. The Ritger–Peppas model was not suitable in describing the release profile of the samples in the reducing condition due to the change in the diffusion layer caused by disulfide bond degradation and swelling.

### 3.9. Cytocompatibility of NanoC

**Figure 11:**
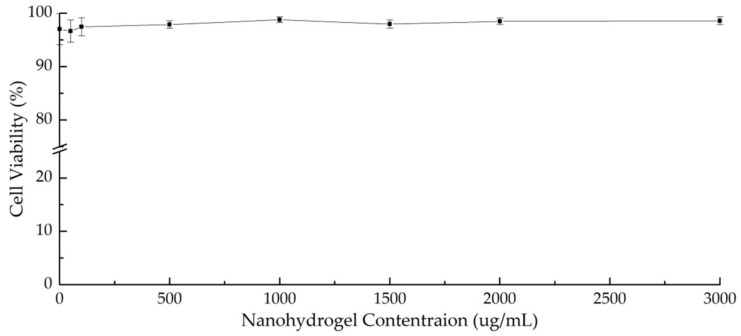
Cytotoxicity results of NanoC by LDH assay in RAW 264.7 cell line.

Cytocompatibility is an essential condition for nanoparticles to become a feasible drug delivery system. Therefore, cell cytotoxicity of nanohydrogels was evaluated against the RAW 264.7 macrophages cell line. This cell line was selected because much one evidence has supported the contribution of macrophages as antigen-presenting cells and as phagocytes taking up nanoparticles. Macrophages have shown a trend to gather around tumor regions and inflamed tissue. Therefore, a tumor delivery route has been studied for internalization of nanoparticles into macrophages by passive targeting [104]. Macrophage cells treated with various concentrations (0–3000 μg/mL) of nanoparticles for 24 h showed similar cell growth to control cells and no significant decrease in the proliferative activity of cells was observed even at the highest concentration employed. Additionally, BSA served as a model drug in this study and has been widely used as a model antigen in immune vaccine studies [105]. Based on the encapsulation efficiency of BSA by NanoC, 530 μg NanoC are needed to delivery 100 μg BSA, which is the suggested amount to induce an immune effect in mice [106,107,108]. According to cytotoxicity results, there were more than 90% cells viable even with NanoC at a high concentration of 3000 μg/mL. It can be concluded that the addition of different concentrations of NanoC to the cell culture did not cause untoward effects on macrophages cells, which confirmed that NanoC can provide significant cytocompatibility (Figure 11). Moreover, according to our previous study, the bulk hydrogel system cross-linked by Trib-sulf and HA-SH was demonstrated to have a significant anti-inflammatory effect in an osteoarthritis mouse model contributing to sustained release of HA during hydrogel degradation [60]. Therefore, we can infer that an anti-inflammatory effect can be considered as one potential benefit of a NanoC delivery system induced by redox-responsive degradation and subsequent HA-SH controlled release in a reducing environment.

### 3.10. Internalization of NanoC into Macrophages

**Figure 12:**
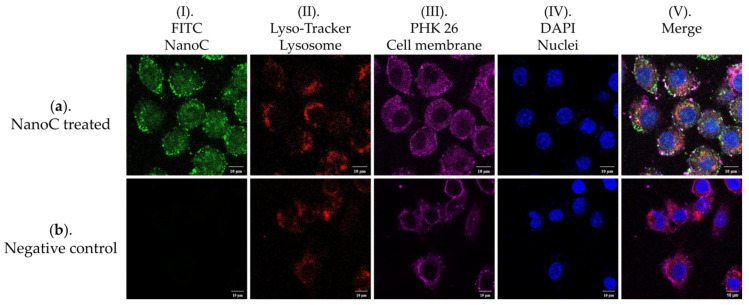
Confocal images of RAW264.7 cells treated by (**a**) FITC-loaded NanoC_72 6 h and (**b**) RAW264.7 without treatment as a negative control. (**I**) NanoC (FITC encapsulated, Green); (**II**) lysosome (Lyso-Tracker-labeled, deep red); (**III**) cell membrane (PKH 26-labeled, red); (**IV**) nuclei (DAPI-labeled, blue); (**V**) merge channel (scale bar = 10 μm).

The RAW 264.7 macrophage cell line was selected as a model cell line to initially discovery the potential of this NanoC delivery system in the field of macrophage-mediated cancer immunotherapy. To visualize NanoC taken up by RAW 264.7 macrophages, the CLSM was employed to further demonstrate NanoC delivery and internalization of macrophages. Figure 12 exhibits confocal microscopy images of the FITC-labeled NanoC treated RAW 264.7 macrophage cell line and the same cell line without NanoC treatment as a negative control in separate single channels and merged multi-channels. The presence of the NanoC was determined in the green fluorescence channel (Chanel I of Figure 12a) due to FITC labeling which was absent in the negative control (Chanel I of Figure 12b). It can be observed from the merged image of the NanoC treated sample (Chanel V of Figure 12a that the FITC labeled NanoCs was present in the entire RAW 264.7 macrophages under the CLSM, which confirmed that the NanoC was efficiently internalized by macrophages. According to the literature, the internalization efficiency of nanoparticles into macrophages can be affected by the component, particle size, surface nature and zeta potential of the nanoparticles [109,110]. Additionally, several studies showed that nanoparticle, as foreign substances, can induce mitosis of macrophages, affect polymerization of macrophages and the cell cycle [111,112,113]. However, in this study, there were no specific differences observed in proliferation between the NanoC-treated RAW 264.7 macrophages and the control group under the CLSM. However, it will be interesting to study the effect of NanoC on the cell cycle of macrophages and also changing of macrophage gene expression, such as the cytokine profile, before and after NanoC uptake, to understand the interaction between the NanoC and the macrophage. Therefore, this internalization result can be considered an instructive preliminary for future studies including evaluation of macrophage uptake of this nanohydrogel system formulated with different physicochemical parameters, internalization by different immune cell lines such as other antigen-presenting cells, and the influence of NanoC on the cell cycle and gene expression of the cell lines, to extend the potential of this nanohydrogel system in cancer immunotherapy.

## 4. Conclusions

In this study, redox responsive NanoC systems possessing a hydrophilic disulfide cross-linked core and an interfacially Michael addition cross-linked hydrophobic shell were successfully developed and studied as potential intracellular delivery systems. It is evident that the NanoC can efficiently protect and retain the activity of the encapsulated model protein in the physiological environment, while rapidly releasing the payload in reducing conditions by redox-triggered degradation. Additionally, NanoC showed excellent cytocompatibility and can be efficiently internalized by macrophages. Therefore, this redox-responsive NanoC holds great potential as a cell-specific and controlled drug release vehicle, with possible applications in the field of macrophage-mediated cancer immunotherapy. Future work will be aimed at determining how effective NanoC is in delivering cancer immunotherapeutics. For example, we will investigate if antigen-presenting cells, such as macrophages or dendritic cells, can successfully cross-present antigens after uptake of protein or peptide-loaded NanoC as cancer vaccines. In addition, further studies should be developed to add specific targeting ligands onto the shell of NanoC in order to achieve an active site-specific intracellular delivery system. It is also significant to investigate the intracellular macromolecule delivery behavior and cell internalization of the nanocapsules *in vivo*. As nanotechnology is gaining increasing attention in the field of medicine, neotype nano-scale delivery systems manufactured via novel technology and materials presenting new structures and smart functions are being developed to promote the clinical study and industrial production of nanomedicine. Therefore, we believe the novel redox-responsive core-shell structure nanohydrogel presented in this study, although with only preliminary physicochemical characterization and evaluation of in vitro cytocompatibility and internalization with a macrophage cell line, still represents exciting potential for further in vitro and in vivo assays, and can provide exciting possibilities for medical and diagnosis applications.

## Figures and Tables

**Table 1:** Experimental variables and test levels under study.

Experimental Variables	Levels		
	1	2	3
A. Homogenization speed (rpm)	8000	10,750	13,500
B. Homogenization time (min)	15	30	45
C. Cross-linking time (hour)	24	48	72

**Table 2:** Experimental 3^3^ factorial design for nanocapsules (NanoC) preparation and the response variables under study.

Experiment Number	Design (Coded Variables)			Plan (Natural Variables)			*Y*_1_	*Y*_2_
	A	B	C	Homogenization		Cross Linking	Particle	PDI
				Speed (rpm)	Time (min)	Time (hour)	Size (nm)	
1	1	1	1	8000	15	24	3509	0.70
2	2	1	1	10,750	15	24	1920	0.52
3	3	1	1	13,500	15	24	1300	0.42
4	1	2	1	8000	30	24	1980	0.33
5	2	2	1	10,750	30	24	865	0.22
6	3	2	1	13,500	30	24	514	0.13
7	1	3	1	8000	45	24	1362	0.38
8	2	3	1	10,750	45	24	402	0.15
9	3	3	1	13,500	45	24	252.5	0.11
10	1	1	2	8000	15	48	2460	0.58
11	2	1	2	10,750	15	48	1259	0.48
12	3	1	2	13,500	15	48	730	0.39
13	1	2	2	8000	30	48	1369	0.20
14	2	2	2	10,750	30	48	215	0.11
15	3	2	2	13,500	30	48	350	0.10
16	1	3	2	8000	45	48	890	0.26
17	2	3	2	10,750	45	48	400	0.10
18	3	3	2	13,500	45	48	270	0.10
19	1	1	3	8000	15	72	2281	0.80
20	2	1	3	10,750	15	72	1088	0.57
21	3	1	3	13,500	15	72	917	0.51
22	1	2	3	8000	30	72	1210	0.44
23	2	2	3	10,750	30	72	300	0.18
24	3	2	3	13,500	30	72	391	0.19
25	1	3	3	8000	45	72	1142	0.43
26	2	3	3	10,750	45	72	309.3	0.29
27	3	3	3	13,500	45	72	251	0.19

**Table 3:** Preparation condition, particle size, polydispersity index (PDI) and zeta potential of the NanoC and nanosphere (NanoS) characterized by dynamic light scattering (DLS).

Abbreviation	Water Phase	Organic Phase	Homogenization		Cross-Linking Time (hour)	Particle Size (nm)	PDI	Zeta Potential (mV)
			Speed (rpm)	Time (min)				
NanoC_24	HA-SH/ Water	Trib-sulf/Lecithin/CHCl_3_	13,500	45	24	247.40 ± 13.33	0.14 ± 0.06	−18.93 ± 1.18
NanoC_48	HA-SH/ Water	Trib-sulf/Lecithin/CHCl_3_	13,500	45	48	265.40 ± 24.20	0.14 ± 0.01	−14.63 ± 1.21
NanoC_72	HA-SH/ Water	Trib-sulf/Lecithin/CHCl_3_	13,500	45	72	251.30 ± 5.73	0.12 ± 0.04	−12.67 ± 0.60
NanoS_24	HA-SH/ Water	Lecithin/CHCl_3_	13,500	45	24	247.40 ± 9.32	0.23 ± 0.02	−28.87 ± 0.72
NanoS_48	HA-SH/ Water	Lecithin/CHCl_3_	13,500	45	48	235.40 ± 4.60	0.22 ± 0.01	−26.00 ± 0.78
NanoS_72	HA-SH/ Water	Lecithin/CHCl_3_	13,500	45	72	233.67 ± 7.40	0.26 ± 0.02	−27.13 ± 2.08

**Table 4:** Assignment of Raman bands of observed in HA-SH polymer, NanoS_72 and NanoC_72.

No.	Raman Shifts (cm-1)				Assignment	
	Measurement			Reference	Bond	Contributed Polymer
	HA-SH Polymer	NanoS_72	NanoC_72			
1	--	--	297	300–400 [86,87]	SO_2 bend_	-vinyl sulfone of Trib-sulf
2	--	--	332	300–400 [86,87]	SO_2 bend_	-vinyl sulfone of Trib-sulf
3	--	496	505	498 [86,88,89]	S-S_str_	disulfide cross-linked core
4	--	561	569	560 [86,88,89]	S-S_bend_	disulfide cross-linked core
5	665	658	666	660 [86]	C-S_str_	linkage of thiol group on HA chain
6	--	-	703	655–704 [86,88,89]	C-S_str_	Michael addition between thiol groups and vinyl sulfone groups
7	889	887	881	889 [81,84,85]	--	HA-SH
8	941	940	938	949 [81,84,85]	--	HA-SH
9	1037	1034	1023	1047 [81,84,85]	C–C_str_ C–O_str_	HA-SH
10	1081	1086	1095	1091 [81,84,85]	C–OH_bend_ acetyl group	HA-SH
11	1118	1114	1111	1125 [81,84,85]	C_(4)_–OH_bend_ C_(4)_–H_bend_	HA-SH
12	1207	1198	1194	1205 [81,84,85]	CH_2twist_	HA-SH
13	1329	1327	1322	1328 [81,84,85]	C–H_bend_ Amide III	HA-SH
14	1374	1366	1365	1372 [81,84,85]	C–H_bend_	HA-SH
15	1409	1405	1415	1406 [81,84,85]	C–N_str_ C–H_def_	HA-SH
16	1645	1648	1644	1660 [81,84,85]	C=C Amide I	HA-SH
17	2656	--	--	2574 [86]	–SH_str_	thiol group of HA-SH
18	2905	2905	2856	2904 [81,84,85]	C–H_str_	HA-SH
19	2933	2933	2903	2933 [81,84,85]	N–H_str_	HA-SH
20	--	--	2975	2964 [90,91]	–CH_2str._	PEG segment of Trib-sulf

**Table 5:** Characterization of protein loaded NanoC and NanoS.

Nanohydrogel	Protein	Particle Size (nm)	PDI	Zeta Potential (mV)	Encapsulation Yield (%)	Loading Capacity (%)
NanoC_72	--	251.30 ± 5.74	0.12 ± 0.04	−12.7 ± 0.60	--	--
	BSA	319.37 ± 13.10	0.21 ± 0.04	−12.3 ± 0.44	93.41 ± 5.49	18.68 ± 1.10
	HRP	246.50 ± 3.16	0.32 ± 0.03	−12.7 ± 0.56	47.81 ± 1.80	9.56 ± 0.36
	CC	321.73 ± 7.95	0.20 ± 0.01	−12.2 ± 0.40	90.78 ± 4.37	18.16 ± 0.87
NanoS_72	--	233.67 ± 7.40	0.26 ± 0.02	−27.1 ± 2.08	--	--
	BSA	334.83 ± 1.76	0.21 ± 0.02	−25.5 ± 0.95	93.28 ± 6.59	18.66 ± 1.32
	HRP	277.27 ± 4.73	0.25 ± 0.03	−26.5 ± 0.76	52.13 ± 4.50	10.43 ± 0.90
	CC	352.10 ± 8.70	0.23 ± 0.03	−26.6 ± 1.56	90.91 ± 3.06	18.18 ± 0.61

**Table 6:** Kinetic parameters of peroxidase-like enzymatic activity assay for free enzymes and CC/HRP loaded NanoC and NanoS.

Nanohydrogel/Protein	*Km* (μM)	*v_max_* (μM/min)	*k_cat_*	*R*^2^
HRP	262.31 ± 14.27	1.59 ± 0.03	19.88 ± 0.03	0.99
NanoC_72_HRP	296.17 ± 16.46	1.41 ± 0.06	17.63± 0.06	0.99
NanoS_72_HRP	347.49 ± 21.28	1.51 ± 0.04	18.88± 0.04	0.99
CC	29.35 ± 4.99	0.27 ± 0.03	0.30 ± 0.03	0.93
NanoC_72_CC	39.12 ± 12.29	0.18 ± 0.01	0.20 ± 0.01	0.94
NanoS_72_CC	46.34 ± 15.79	0.20 ± 0.01	0.22 ± 0.01	0.95

## Data Availability

Not applicable.

## Supplementary Materials

The following are available online at https://www.mdpi.com/1999-4923/13/12/2048/s1. Figure S1: Synthesis pathway of vinyl sulfonated poly(HPMAm-lactate_1-2_)-PEG-poly(HPMAm-lactate_1-2_) triblock copolymer (Trib-sulf). Figure S2: Synthesis pathway of thiolated hyaluronic acid (HA-SH). Figure S3: ^1^H-NMR spectrum of vinyl sulfonated triblock copolymer in DMSO. Figure S4: ^1^H-NMR spectrum of HA-SH in in D_2_O. Table S1: Characteristics of synthesized Trib and Trib-sulf copolymers. Table S2: Parameter according to different kinetic models for protein release from NanoC and NanoS in PBS. Table S3: Parameter according to different kinetic models for protein release from NanoC and NanoS in GSH.

## Abbreviations

3^3^	three-level full factorial design
[*S*]	substrate concentration
^1^H-NMR	proton nuclear magnetic resonance
ABTS	2,2′-azinobis-(2-ethylbenzthiazoline-6-sulfonate)
BCA	bicinchoninic acid
BSA	bovine serum albumin
CC	cytochrome c
CHCl_3_	chloroform
CLSM	confocal laser scanning microscopy
DAPI	4′,6-diamidino-2-phenylindole
DLS	dynamic light scattering
DMEM	Dulbecco’s modified eagle medium
DOE	design of experiments
DPTS	4-(dimethylamino) pyridinium-4-toluenesulfonate
DS%	substitution degree
EE	encapsulation efficiency
*FCS*	fetal calf serum
FE-SEM	field emission-scanning electron microscope
FITC	fluorescein isothiocyanate
FT-IR	Fourier transform infrared spectroscopy
GPC	gel permeation chromatography
HA-SH	thiolated hyaluronic acid
HRP	horseradish peroxidase
*K_m_*	Michaelis constant
LC	loading capacity
LCST	lower critical solution temperature
LDH	lactate dehydrogenase
Mn	number average molecular weight
Mw	molecular weight
NanoC	nanocapsule
NanoS	nanosphere
OV	oncolytic virus
PBS	phosphate buffered saline
PDI	polydispersity index
PS	penicillin-streptomycin
RS	Raman spectroscopy
SEM	Scanning electron microscopy
TEM	Transmission electron microscopy
Trib-sulf	vinyl sulfonated poly(n-(2-hydroxypropyl) methacrylamide mono/di lactate)-polyethylene glycol copolymer
*v*	initial rate of the reaction
*V_max_*	maximum rate of the reaction
W/O	water in oil
