# Supplementary material for: Dually Cross-Linked Core-Shell Structure Nanohydrogel with Redox–Responsive Degradability for Intracellular Delivery
Source: Pharmaceutics. 2021 Nov 30;13(12):2048. doi: 10.3390/pharmaceutics13122048 (PMC8708258; doi:10.3390/pharmaceutics13122048)
Supplement: Supplementary file 1 [file pharmaceutics-13-02048-s001.zip › pharmaceutics-1450311-supplementary.pdf]

pharmaceutics

Supplementary Materials: Dually Cross-Linked Core-Shell Structure Nanohydrogel with Redox–Responsive Degradability for Intracellular Delivery

Siyuan Deng, Maria Rosa Gigliobianco, Yimin Mijiti, Marco Minicucci, Manuela Cortese, Campisi Barbara, Dario Voinovich, Michela Battistelli, Sara Salucci, Pietro Gobbi, Giulio Lupidi, Giorgia Zambito, Laura Mezzanotte, Roberta Censi and Piera Di Martino

1. Synthesis of Vinyl Sulfonated Triblock Copolymer (Trib-sulf)

N-(2-hydroxypropyl) methacrylamide (HPMAm) was synthesized by Schotten-Baumann reaction of 1-amino-2 propanol and methacryloyl chloride according to the work of Tucker et al with a yield of 88.90%. [1]

HPMAm-lactate$_1$ and HPMAm-lactate$_2$ were synthesized by ring-opening polymerization of L-lactide with previous synthesized HPMAm in the presence of tin(II) 2-ethylhexanoate stannous octoate ($SnOct_2$) and 4-methoxyphenol (MeHQ) as catalyst and polymerization inhibitor, respectively, according to the procedure modified from Neradovic et al.[2] HPMAm-lactate$_1$ and HPMAm-lactate$_2$ were isolated from a mixture of crude oligomers products performed by a VersaFlash® column (Supelco Merck, Darmstadt, Germany) with a total yield of 39.58% which were used as monomers for the subsequent synthesis of triblock copolymer.

(Polyethylene glycol-4-4′-azobis (4-cyanopentanoic acid))$_n$ (PEG-ABCPA)$_n$ macroinitiator was synthesized by 4-(dimethylamino)pyridinium 4-toluenesulfonate/N,N′-dicyclohexylcarbodiimide (DPTS/DCC) coupling reaction of polyethylene glycol (PEG, 10,000 g/mol) and 4-4′-azobis (4-cyanopentanoic acid) (ABCPA) according to the procedure described by Censi et al.[3] The synthesized (PEG-ABCPA)$_n$ macroinitiator produced with a yield at 79.30%, displayed number average molecular weight (Mn), weight average molecular weight (Mw), and the polydispersity index (PDI) values of 70 kDa, 90 kDa and 1.4, respectively, recorded by gel permeation chromatography (GPC). The proton nuclear magnetic resonance (^{1}H-NMR) result reveals that 1.75 units of ABCPA were present per PEG$_{225}$ chain.

Thermosensitive ABA type poly(HPMAm-lactate$_{1\text{-}2}$)-PEG-poly(HPMAm-lactate$_{1\text{-}2}$) triblock copolymer (Trib) was synthesized by free radical polymerization using HPMAm-lactate$_1$ and HPMAm-lactate$_2$ as thermo-sensitive terminal group and (PEG-ABCPA)$_n$ as macroinitiator.[4] The Trib copolymer produced with a yield at 77.50%, displayed Mn, Mw and PDI values of 36 kDa, 46 kDa and 1.3, respectively, recorded by GPC measurement.

Trib-sulf was synthesized by introducing vinyl sulfone moieties into the previous synthesized Trib copolymer as Michael addition reaction sites, according to the synthesis procedure modified from Dubbini et al.[5] The reaction has been carried out by using one pot synthesis in the presence of DPTS/DCC as catalyst/coupling reagent under water- and oxygen-free conditions, and the synthesis route is reported in Figure S1. The percentage of free hydroxyl groups substituted by vinyl sulfonated groups is defined as substitution degree (DS). The aimed DS was 10% DS. As a first synthesis step, 3-mercaptopropionic acid (3-MPA) (0.184 g, 1.73 mmol) was reacted with divinyl sulfone (DVS) (4.088 g, 34.60 mmol) in 29 mL anhydrous dimethylsulfoxide (DMSO) at room temperature for 4 hours under N_2 atmosphere. Subsequently, another reaction mixture was prepared by dissolving the previously synthesized Trib (2.9 g, 0.0686 mmol), DCC (0.536 g, 2.60 mmol), and DPTS (0.0653 g, 0.260 mmol) in 29 mL anhydrous DMSO under N_2 atmosphere. Once the Trib copolymer was completely dissolved, the 3-MPA/DVS reaction mixture was added dropwise. The reaction was conducted 24 hours at room temperature. The resulting

product was purified by filtration to remove the by-product dicyclohexyl urea (DCU) and dialyzed (Mw cutoff = 12 – 24 kDa) against deionized water at 4 ºC for 3 days. The obtained DS% and Mn of Trib-sulf copolymer were determined ^{1}H-NMR in deuterated dimethyl sulfoxide (DMSO-*d6*). Mn, Mw and PDI were measured by GPC. LCST was characterized by dynamic light scattering (DLS).

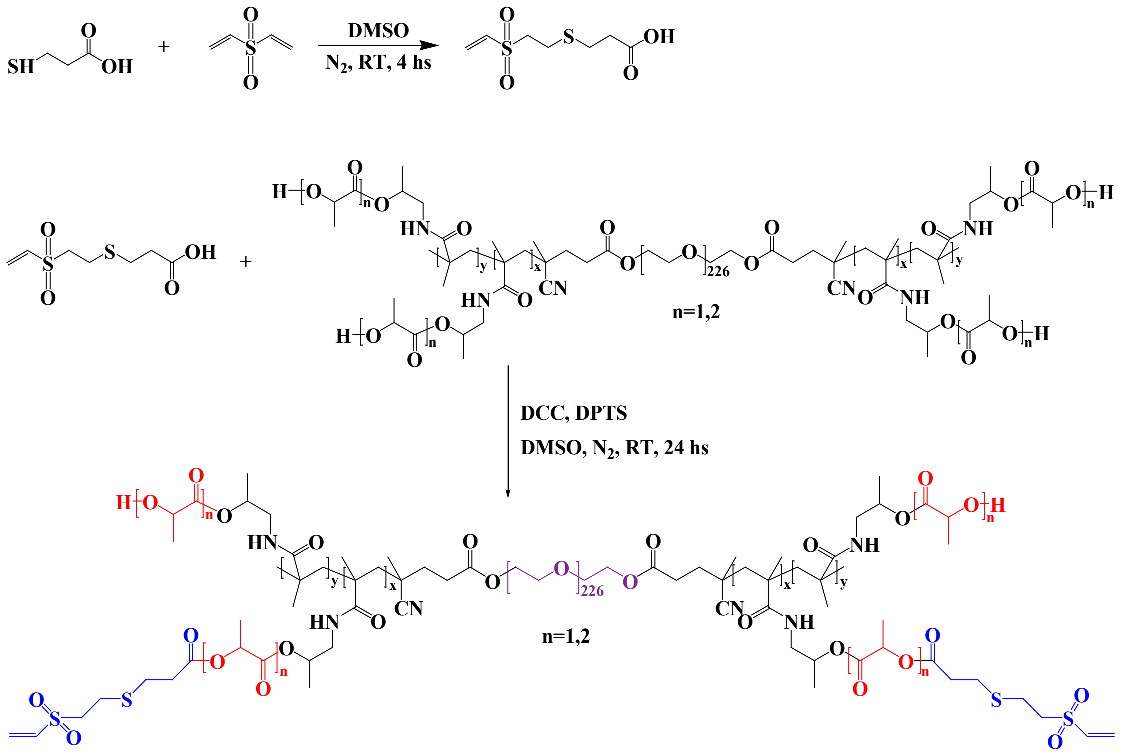

Figure S1. Synthesis pathway of vinyl sulfonated poly(HPMAm-lactate$_{1\text{-}2}$)-PEG-poly(HPMAm-lactate$_{1\text{-}2}$) triblock copolymer (Trib-sulf).

2. Synthesis of Thiolated Hyaluronic Acid (HA-SH)

The synthesis of HA-SH was performed by slightly modifying the procedure described in our previous study.[6] The synthetic scheme of HA-SH is depicted in Figure S2. Firstly, HA was coupled with 3,3′-dithiobis (propanoic dihydrazide) (DTP) by carbodiimide chemistry, and then the disulfide bonds were reduced by dithiothreitol (DTT) to obtain free thiol groups. The number of thiol groups substituted per 100 disaccharide units is defined as substitution degree (DS). Briefly, HA (1.20 g, 0.0317 mmol) reacted with DTP (294.65 mg, 1.235 mmol) in 120 mL ultrapure water at room temperature and pH 4.75. Then, 1-ethyl-3-(3-dimethylaminopropyl) carbodiimide (EDC·HCl, 236.98 mg, 1.235 mmol) was added as a carboxyl activating agent. The reaction was carried out for 48 hours and then stopped by increasing the pH to 7. Subsequently, DTT (4.88 g, 0.0317 mmol) was added to the reaction mixture as reducing agent and the pH was adjusted to 8.5. After 24 hours, the pH was lowered to 3.5. The reaction solution was purified by dialysis (Mw cutoff = 12 – 24 kDa) against 100 mM NaCl solution at 4 ºC, pH 3.5 for 3 days, then dialyzed against deionized water for additional 24 hours. The final product was isolated as dry powder by lyophilization (Freeze dryer, FreeZone, Labconco, Kansas City, United States) and stored at −20 ºC. Thirty percent DS was obtained and characterized by ^{1}H-NMR in D_2O.

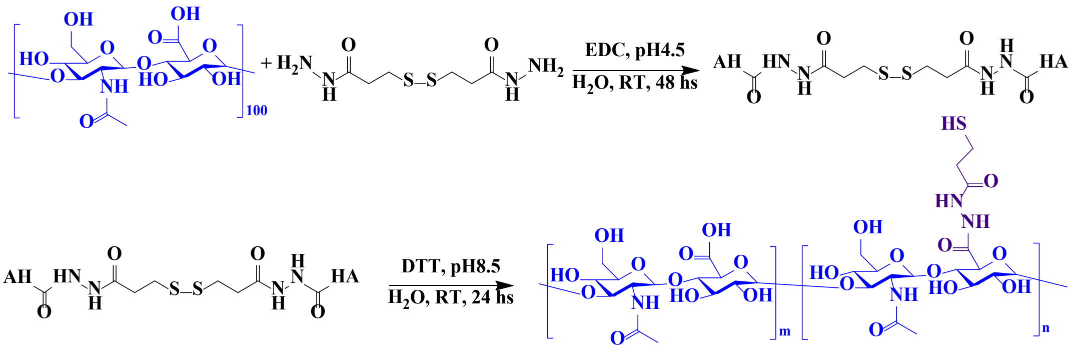

Figure S2. Synthesis pathway of thiolated hyaluronic acid (HA-SH).

3. Proton Nuclear Magnetic Resonance (^{1}H-NMR)

The chemical structures and number-average molecular weight (Mn) of the synthesized polymers were characterized by ^{1}H-NMR (Varian Mercury plus 400, Crawley, UK) using DMSO-*d*$_6$, deuterated chloroform ($CDCl_3$) or D_2O as solvents. Chemical shift was referred to the solvent peak (δ = 2.50 ppm for DMSO-*d*$_6$, δ = 7.26 ppm for $CDCl_3$, δ = 4.79 ppm for D_2O).

4. Gel Permeation Chromatography (GPC)

Gel permeation chromatography was employed to determine Mn, Mw, and the PDI of synthesized polymers. The GPC measurements were carried out by using a TSKgel® G4000Hhr column (Tosoh Bioscience, Tokyo, Japan), ID 7.8 mm × L 30 cm, pore size 5 µm. A series of PEGs with defined molecular weight from 106 to 1039000 Da were used as calibration standards. The eluent was tetrahydrofuran (THF), the elution rate was 1.0 mL/min, and the column temperature was 35 ºC. Samples were dissolved in THF at a concentration of 5 mg/ml.

5. Determination of the Lower Critical Solution Temperature (LCST)

The LCST of thermo-sensitive polymers was determined by DLS using a Zetasizer Nano-S90 (Malvern Panalytical, Worcestershire, UK). The thermo-sensitive copolymer was dissolved in ultrapure water at a concentration of 5 mg/mL. The light scattering intensity was measured under temperature increasing from 5 to 40 ºC, at a heating rate of 1 ºC/min. The LCST refers to the onset temperature of the increase in light scattering intensity.

6. Characterizations of Synthesized Trib-sulf and HA-SH

Vinyl sulfone groups were introduced into triblock copolymer by partial modification of the terminal free hydroxyl groups of HPMAm-lactate$_{1\text{-}2}$ in order to act as crosslinkable moieties for the combination between Trib-sulf and HA-SH during NanoC formulation. Table S1 summarized the main characteristics of the Trib-sulf copolymer before and after the vinyl sulfonation. The Mn of the synthesized Trib-sulf was 41 kDa by ^{1}H-NMR which was higher than the Mn achieved by GPC measurement. It was explained in previous study that GPC measurement used PEG as standard which displayed higher hydrodynamic volume compared with Trib-sulf in the eluent THF.[3] By comparing GPC analysis results of Trib and Trib-Sulf, it turned out that upon the partial modification of free hydroxyl groups with vinyl sulfone groups, the polymers' Mn, Mw and PDI values remained constant, indicating that no degradation of the triblock copolymer occurred during DCC coupling reaction, purification and lyophilization procedures. The chemical structure of Trib-sulf was determined by ^{1}H-NMR and the spectrum displayed in Figure S3. The peak at 3.5 ppm corresponded to the –CH_2– of central PEG segment. The peaks at 4.12-4.22 and 4.80-4.96 were contributed from methine groups of HPMAm-lactate$_{1\text{-}2}$ ending segments. The derivatization of triblock copolymer with vinyl sulfone groups was confirmed by the peaks at 6.23 ppm and 6.98 ppm, which belong to the protons from vinyl

sulfone groups. The peak at 5.3-5.4 ppm was attributed to the terminal hydroxyl group of un-derivated Trib chains. The DS% of vinyl sulfone groups calculated based on ^{1}H-NMR spectrum and Equation S1 was 12%, a value close to the aimed DS% of 10% (Figure S3). The LCST of Trib-sulf was 31 ºC, that was slightly lower than that of Trib, as a consequence of the increased hydrophobicity of the polymer after vinyl sulfonation.[7]

$$DS_{-sulf}\% = \frac{\int I_{6.2}/2}{\int I_{6.2}/2 + \int I_{5.3-5.4}} \times 100\% \qquad (S1)$$

Table S1. Characteristics of synthesized Trib and Trib-sulf copolymers.

Name	Mn(kDa)	Mw(kDa)	PDI	DS%	CP(°C)	Yield(%)
Trib	36[1]<br>44[2]	46[a]	1.3[1]	N.A.	35[3]	77.50
Trib-sulf	37[1]<br>41[2]	47[a]	1.4[1]	12[2]	31[3]	96.60

[1] Determined by GPC; [2] Determined by ^{1}H-NMR; [3] Determined by DLS

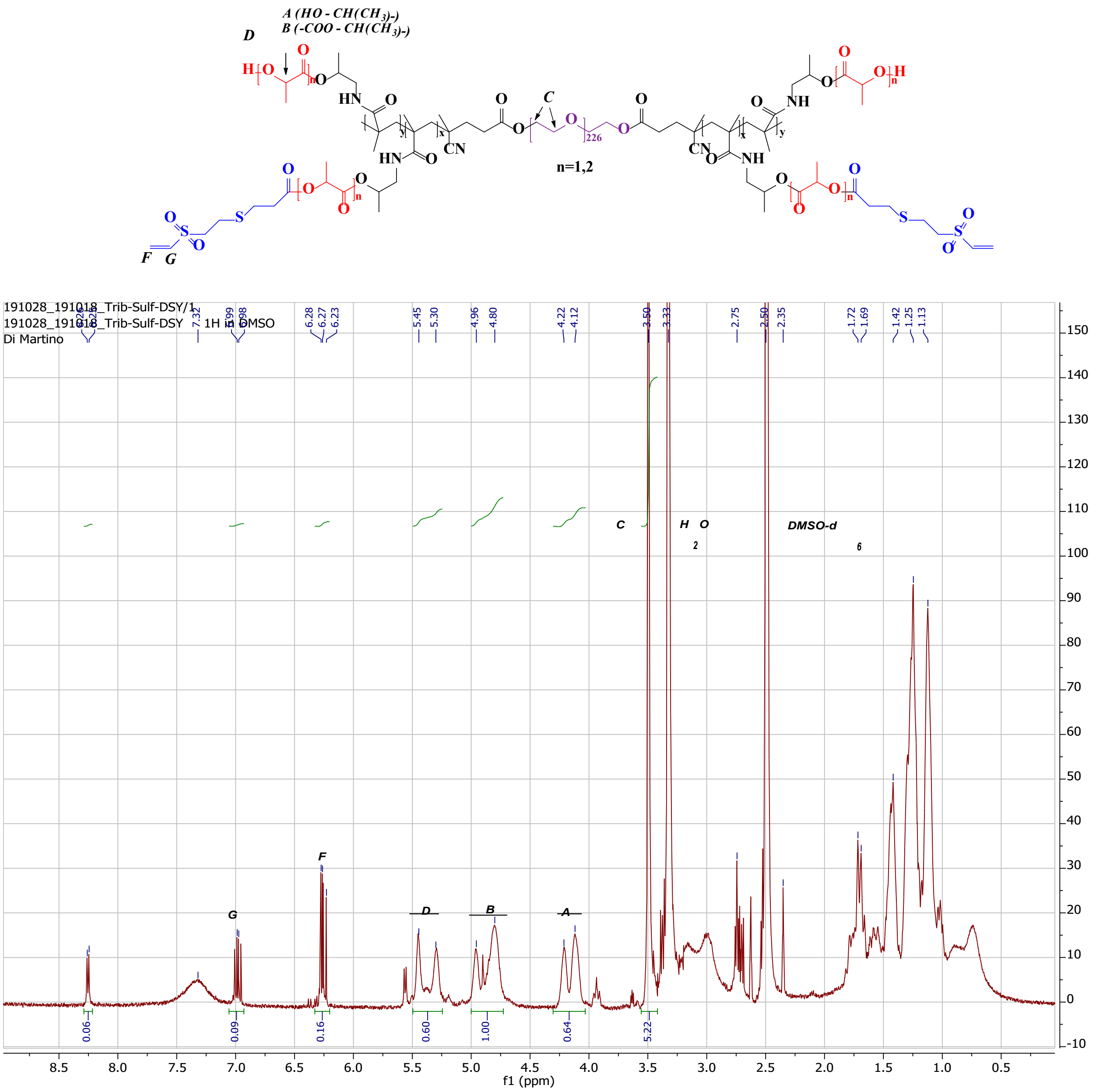

Figure S3. ^{1}H-NMR spectrum of vinyl sulfonated triblock copolymer in DMSO, δ in ppm: 1.15 -1.72 (main chain protons and -C<u>H</u>$_3$ of HPMAm-lactate$_{1-2}$); 3.50(904H,-C<u>H</u>$_2$C<u>H</u>$_2$O-); 4.12-4.22 (1H, -C<u>H</u>(OH)CH$_3$);4.80-4.96 (1H for HPMAm-lactate$_1$,

2H for HPMAm-lactate2 -OC$\underline{H}$(CH_3)COO- and -OC$\underline{H}$(CH_3)CH_2-); 5.30-5.45 (1H, -CH(O$\underline{H}$)CH_3); 6.23-6.28 (2H, -SO_2CH=C$\underline{H_2}$); 6.98-6.99 (-SO_2C$\underline{H}$=CH_2).

HA-SH was synthesized with a quantitative yield of 99.80%. Figure S4 presented the ^{1}H-NMR spectrum of HA-SH. The multi peaks in the range of 3.36 – 4.56 ppm correspond to the main change of HA. The DS% was calculated according to the ^{1}H-NMR spectrum. The two peaks at 2.67 and 2.85 ppm were characteristics of the two methylene groups adjacent to the grafted thiol groups, while the methyl group presented on each repetitive unit of HA-SH was detected at 2.03 ppm and used as internal standard for the calculation of thiolated DS% (Equation S2), the value of which was 27%.

$$DS_{-SH}\% = \frac{3 \times \int (I_{2.67} + I_{2.85})}{4 \times \int I_{2.03}} \quad \text{(S2)}$$

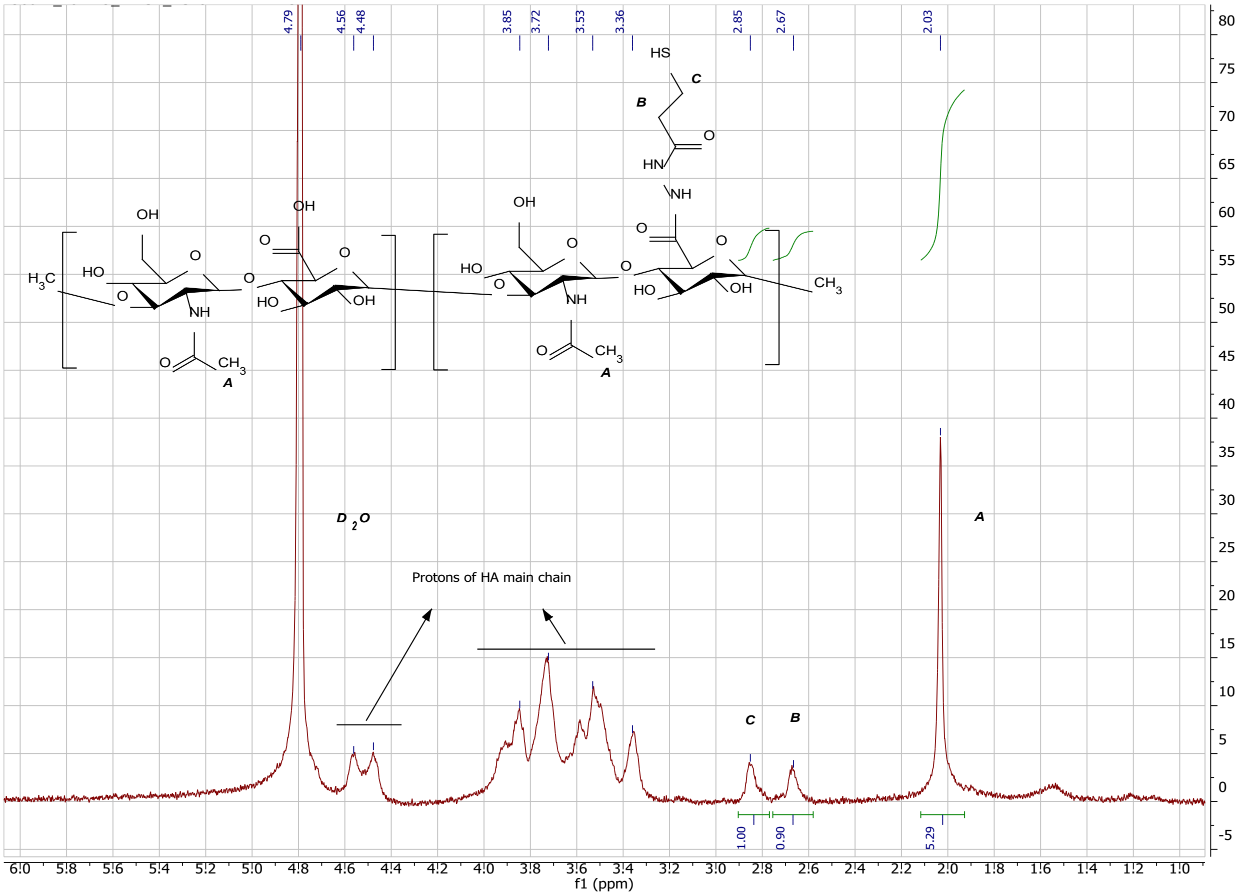

Figure S4. ^{1}H-NMR spectrum of HA-SH in in D_2O, δ in ppm: 2.03 (3H, -NHC(=O)C$\underline{H_3}$); 2.67(2H, -C$\underline{H_2}$$CH_2$SH); 2.85 (2H,-$CH_2C\underline{H_2}$SH); 3.36-4.56 (protons of HA main chains).

7. Kinetic Model for Protein Release of NanoC and NanoS

The experimental protein release data were fitted into Zero order, First order and Ritger–Peppas models. The description of each kinetic model equation is explained as follow [8]:

Zero-order model $$C_t = C_0 + K_0 t \quad \text{(S3)}$$

C_t: amount of drug released at time t

C_0: initial concentration of drug at t = 0

K_0: zero-order rate constant

First-order model $$LogC = LogC_0 + Kt/2.303 \quad \text{(S4)}$$

C: amount of drug remaining at time t

C_0: initial concentration of drug at t = 0

K: First-order rate constant

Ritger Peppas model $$M_t/M_\infty = Kt^n \quad \text{(S5)}$$

M_t/M_∞: Fractional release of the entrapped protein

K: First-order rate constant

n: Diffusional exponent

Table S2. Parameter according to different kinetic models for protein release from NanoC and NanoS in PBS

Name	Protein	Parameters & R^2 of Kinetic Models		
		**Zero-Order**	**First-Order**	**Ritger–Peppas**
NanoC_72	BSA	$K_0 = 4.36\times10^{-4}$, $R^2 = 0.98$	K = 0.035, $R^2 = 0.68$	n = 0.42, K = 8.33×10^{-3}, $R^2 = 0.91$
	HRP	$K_0 = 1.90\times10^{-3}$, $R^2 = 0.95$	K = 0.024, $R^2 = 0.98$	n = 0.51, K = 0.023, $R^2 = 0.92$
	CC	$K_0 = 3.42\times10^{-4}$, $R^2 = 0.79$	K = 0.13, $R^2 = 0.91$	n = 0.26, K = 0.19, $R^2 = 0.95$
NanoS_72	BSA	$K_0 = 0.069$, $R^2 = 0.84$	K = 0.038, $R^2 = 0.92$	n = 0.42, K = 0.039, $R^2 = 0.99$
	HRP	$K_0 = 1.79\times10^{-4}$, $R^2 = 0.91$	K = 0.029 , $R^2 = 0.95$	n = 0.51, K = 0.030, $R^2 = 0.99$
	CC	$K_0 = 4.7\times10^{-4}$, R2 = 0.74	K = 0.13, $R^2 = 0.86$	n = 0.25, K = 0.24, $R^2 = 0.94$

Table S3. Parameter according to different kinetic models for protein release from NanoC and NanoS in GSH

Name	Protein	Parameters & R^2 of Kinetic Models		
		Release data of 1-14 hours/Zero-Order	Release data of 14-150 hours/First-Order	Release data of 14-150 hours/ Ritger–Peppas model
NanoC_72	BSA	$K_0 = 0.022$, $R^2 = 0.99$	K = 0.041, $R^2 = 0.99$	n = 0.32, K = 0.19, $R^2 = 0.94$
	HRP	$K_0 = 0.025$, $R^2 = 0.99$	K = 0.045, $R^2 = 0.98$	n = 0.22, K = 0.32, $R^2 = 0.87$
	CC	$K_0 = 0.074$, $R^2 = 0.96$	K = 0.11, $R^2 = 0.95$	n = 0.18, K = 0.42, $R^2 = 0.93$
NanoS_72	BSA	$K_0 = 0.039$, $R^2 = 0.93$	K = 0.063, $R^2 = 0.98$	n = 0.18, K = 0.41, $R^2 = 0.86$
	HRP	$K_0 = 0.038$, $R^2 = 0.98$	K = 0.080, $R^2 = 0.98$	n = 0.16, K = 0.46, $R^2 = 0.81$
	CC	$K_0 = 0.067$, $R^2 = 0.96$	K = 0.11, $R^2 = 0.97$	n = 0.15, K = 0.47, $R^2 = 0.92$

References

1. Tucker, B.S.; Stewart, J.D.; Aguirre, J.I.; Holliday, L.S.; Figg, C.A.; Messer, J.G.; Sumerlin, B.S. Role of Polymer Architecture on the Activity of Polymer–Protein Conjugates for the Treatment of Accelerated Bone Loss Disorders. *Biomacromolecules*, **2015**. *16*, 2374–2381, doi:10.1021/acs.biomac.5b00623.
2. Neradovic, D.; Van Steenbergen, M.; Vansteelant, L.; Meijer, Y.; Van Nostrum, C.; Hennink, W. Degradation mechanism and kinetics of thermosensitive polyacrylamides containing lactic acid side chains. *Macromolecules*, **2003**. *36*, 7491–7498, doi:10.1021/ma034381n.
3. Censi, R.; Schuurman, W.; Malda, J.; Di Dato, G.; Burgisser, P.E.; Dhert, W.J.; Van Nostrum, C.F.; Di Martino, P.; Vermonden, T.; Hennink, W.E. A printable photopolymerizable thermosensitive p (HPMAm‐lactate)‐PEG hydrogel for tissue engineering. *Adv. Funct. Mater.*, **2011**. *21*, 1833–1842, doi:10.1002/adfm.201002428.
4. Soga, O.; van Nostrum, C.F.; Ramzi, A.; Visser, T.; Soulimani, F.; Frederik, P.M.; Bomans, P.H.; Hennink, W.E. Physicochemical characterization of degradable thermosensitive polymeric micelles. *Langmuir*, **2004**. *20*, 9388–9395, doi:10.1021/la048354h.

5. Dubbini, A.; Censi, R.; Butini, M.E.; Sabbieti, M.G.; Agas, D.; Vermonden, T.; Di Martino, P. Injectable hyaluronic acid/PEG-p (HPMAm-lac)-based hydrogels dually cross-linked by thermal gelling and Michael addition. *Eur. Polym. J.*, **2015**. *72*, 423–437, doi:10.1016/j.eurpolymj.2015.07.036.
6. Agas, D.; Laus, F.; Lacava, G.; Marchegiani, A.; Deng, S.; Magnoni, F.; Silva, G.G.; Di Martino, P.; Sabbieti, M.G.; Censi, R. Thermosensitive hybrid hyaluronan/p (HPMAm‐lac)‐PEG hydrogels enhance cartilage regeneration in a mouse model of osteoarthritis. *J. Cell Physiol.*, **2019**. *234*, 20013–20027, doi:10.1002/jcp.28598.
7. Censi, R.; Vermonden, T.; van Steenbergen, M.J.; Deschout, H.; Braeckmans, K.; De Smedt, S.C.; van Nostrum, C.F.; Di Martino, P.; Hennink, W. Photopolymerized thermosensitive hydrogels for tailorable diffusion-controlled protein delivery. *J. Control. Release*, **2009**. *140*, 230–236, doi:10.1016/j.jconrel.2009.06.003.
8. Laracuente, M.-L.; Marina, H.Y.; McHugh, K.J.J.J.o.C.R. Zero-order drug delivery: State of the art and future prospects. **2020**.
